# Causal insights into immune cell profiles, plasma metabolites, and bladder cancer: A Mendelian randomization approach

**DOI:** 10.1097/MD.0000000000046062

**Published:** 2025-11-21

**Authors:** Juan Yang, Zhangxiao Xu, Bo Tao, Yuan Zhao, Yiran Ma, Yuanjian Niu, Yunpeng He, Jian Wu, Lijun Wang, Zitao Zhong

**Affiliations:** a Faculty of Life Science and Technology & The Affiliated Anning First People’s Hospital, Kunming University of Science and Technology, Kunming, China; b Faculty of Life Science and Technology, Kunming University of Science and Technology, Kunming, Yunnan, China.

**Keywords:** bladder cancer, causal relationship, immune cells, Mendelian randomization, plasma metabolites

## Abstract

This study aimed to investigate the causal associations between immune cells, plasma metabolites, and bladder cancer (BC) using Mendelian randomization (MR). Utilizing summary data from genome-wide association studies, the study examined the causal effects among 731 immune cell phenotypes, 1400 plasma metabolites, and BC. Bidirectional MR analysis was conducted to assess the links between immune cells and BC, and complemented by two-step mediation analysis and multivariate MR to identify potential mediating metabolites. Sixteen immune cell phenotypes were found to have causal associations with BC, including one with reverse causality. Additionally, 48 metabolites were identified as being causally associated with BC. Through two-step and multivariate MR analyses, 5 immune cell phenotypes and 5 plasma metabolites were identified as mediators in the causal pathway to BC. CD4+ activated cells was causally associated with BC, mediated by the unknown metabolite X-12730, with the highest mediation ratio of 11.1% CD4/CD8br showing a causal relationship with BC, mediated by choline levels, with a mediation ratio of 8.78%. CD19 on IgD− CD24− associated with BC, mediated by 4-vinylphenol sulfate levels, with a mediation ratio of 10.9%. CD19 on IgD− CD27− was linked to BC, mediated by ribitol levels, with a mediation ratio of 6.58%. CD38 on IgD+ CD24− B cells was associated with BC, mediated by pimeloylcarnitine/3-methyladipoylcarnitine (C7-DC) levels, with a mediation ratio of 6.73%. This study identified 5 immune cell phenotypes that are causally associated with BC, mediated through 5 plasma metabolites. These findings enhance our understanding of the biological pathways involved, provide potential biomarkers for identifying at-risk populations, and offer insights into strategies for early prevention and diagnosis of BC.

## 1. Introduction

Bladder cancer (BC), also referred to as urothelial carcinoma, is a malignancy arising from the cells that line the bladder.^[1]^ According to research statistics, BC is ranked 4th in cancer incidence among men, with 6% of newly diagnosed cancer cases being BC, in addition to its significant impact on patients’ quality of life, morbidity, mortality, and healthcare system. Bladder cancer development is influenced by a variety of internal and external factors, including genetic susceptibility and the environment; however, its specific etiology and pathogenesis are still not fully understood.

The tumor microenvironment (TME) constitutes the overall complex ecosystem of the tumor, containing tumor cells, stromal cells, immune cells, metabolic products, and secreted proteins. Recent studies have demonstrated that metabolic reestablishment within the TME plays a key role in suppressing tumor immunity. For example, overproduction of metabolic byproducts disrupts the metabolic reprogramming of T cells, thereby weakening their antitumor capacity. A growing number of studies have emphasized the critical role of TME in the development and progression of BC. Therefore, it is crucial to delve in depth into study the immune cells and metabolites affecting the risk of BC, which is important for elucidating the immune microenvironment in BC progression mechanisms.

Mendelian randomization (MR) is a term applied to the use of genetic variation to address the causal question of how modifiable exposures affect different outcomes. The rationale for MR uses genetic variation, known as single nucleotide polymorphisms (SNPs), as an instrumental variable (IV) to determine whether the observed association between a risk factor and outcome is consistent with a causal effect.^[2]^ Recent MR studies have demonstrated that immune cell-associated SNPs can serve as exposure factors that influence tumor development. However, the relationship between immune cells, metabolites, and BC has not yet been explored using MR.

This study employs mediated Mendelian randomization to systematically evaluate the causal relationships among immune cells, plasma metabolites, and BC, with a particular focus on the potential mechanisms by which immune cells influence the development and progression of BC through metabolic mediation. The findings of this research may offer novel theoretical insights for the prevention, early diagnosis, and targeted treatment of BC, and provide new directions for etiological investigations and clinical translational research.

## 2. Materials and methods

### 2.1. Research design

**Figure 1.**
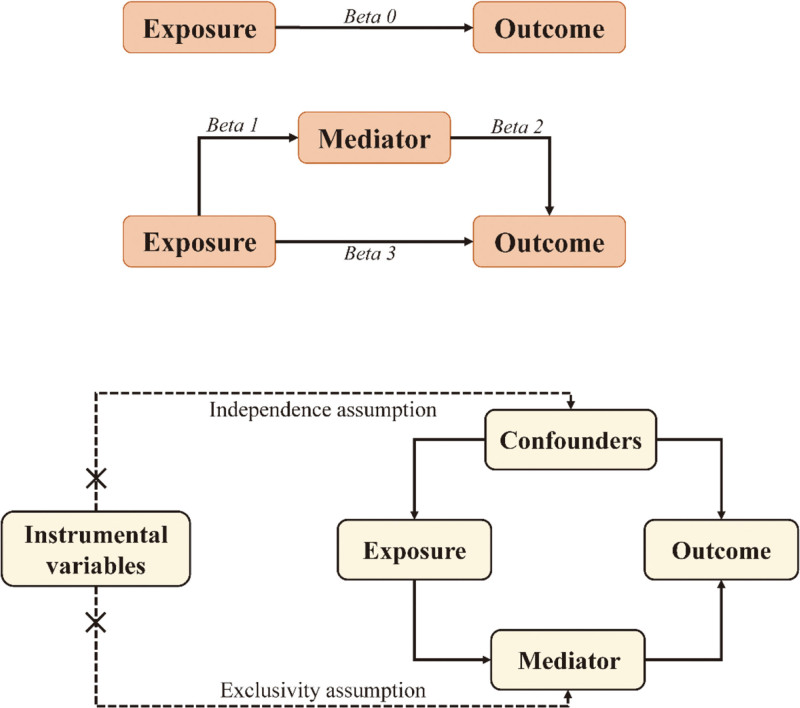
(A) Mediation analysis. (B) Conditions satisfied by instrumental variables.

This study investigates the causal relationship between 731 immune cell phenotypes and BC using bidirectional MR analysis and mediation analysis based on 2 independent samples. It also explores the potential regulatory role of plasma metabolites in this relationship. First, BC was designated as the primary outcome, while the 731 immune cell phenotypes were identified as potential exposures, allowing for a comprehensive investigation of the causal relationship between immune cell phenotypes and BC. Subsequently, we examined the causal relationships between 1400 plasma metabolites and both BC and immune cell phenotypes, evaluating their respective mediation effects (Fig. 1A).The IVs used in this study must satisfy 3 key assumptions (Fig. 1B): genetic variants are directly associated with the exposure, genetic variants are independent of potential confounders affecting both exposure and outcome, and genetic variants do not influence the outcome through pathways other than the exposure.

### 2.2. Dataset collection

#### 2.2.1. Source of the BC genome-wide association studies (GWAS) dataset

Statistical summary data for BC GWAS were obtained from the Medical Research Council Integrative Epidemiology Unit OpenGenome-Wide Association Studies database (Medical Research Council Integrative Epidemiology Unit OpenGWAS) (https://gwas.mrcieu.ac.uk/), UK. This website provides databases and tools for GWAS. We selected 9904,926 SNPs, including 1279 BC cases and 372,016 healthy controls (ID: ieu-b-4874).

#### 2.2.2. GWAS data on immune cell phenotypes

We obtained generalized GWAS statistics for immune cells from the MRCEU open database (accession numbers ranging from GCST 90001391 to GCST 90002121). These data were obtained from 3757 individuals of European ancestry and encompass approximately 22 million SNPs. This GWAS investigated the complex genetic regulation of immune cells in autoimmune diseases.^[3]^ These data covered 731 immune cells with different traits, including 118 absolute counts (AC), 389 median fluorescence intensities, 32 morphological parameters, 192 relative counts, and their association with 22 million SNPs.

#### 2.2.3. GWAS data on metabolites

We assessed metabolite GWAS statistics to study their mediating effects. Generalized GWAS statistics for metabolites were retrieved by searching the GWAS Catalog with accession numbers ranging from GCST90199621 to GCST90201020. The data were derived from the Canadian Longitudinal Study on Aging, including a total of 8299 individuals of European ancestry. This study analyzed 1400 metabolic traits, comprising 1091 plasma metabolites and 309 metabolite ratios. Among the plasma metabolites, 850 identified compounds were classified into 8 major super pathways: lipids, amino acids, xenobiotics, nucleotides, cofactors and vitamins, carbohydrates, peptides, and energy metabolism. The remaining 241 metabolites were categorized as unknown compounds.^[4]^

### 2.3. Mendelian randomization analysis

#### 2.3.1. Identification of IVs

To identify IVs that significantly influence BC risk, we implemented the following strategy: SNPs were selected based on their correlation with exposure (immune cells or metabolites) using the following criteria: *P* < 1e−5, a distance greater than or equal to 10,000 kb, and exclusion of SNPs with chain disequilibrium (*r*^2^ < 0.001). This procedure yielded 18,621 SNPs for immune cells and 34,843 SNPs for metabolites. When the *P* threshold was set as 5e−8, too few SNPs were obtained to support further studies. Thus, we set the *P* threshold to 1e−5 by referring to the criteria in previous studies.^[5–7]^

In addition, the *F*-test was used to eliminate weak IVs with a threshold of 10. SNPs with an *F*-value <10 were excluded.^[8]^ The *F*-value was calculated as follows:


$$\begin{array}{l} F=R^{2}\left(N-2\right)/\left(1-R^{2}\right) \end{array}$$

where *R*^2^ is the variance and N is the sample size of the exposed data. The formula for calculating *R*^2^ is as follows:


$$\begin{array}{l} R^{2}=2\times \left(1-MAF\right)\times MAF\times \beta^{2}, \end{array}$$

where minor allele frequency indicates the effect size of exposure. By implementing these filtering criteria, we aimed to select informative and reliable IVs for the MR analysis.

#### 2.3.2. Mendelian randomization analysis

MR analysis was performed to assess the causal relationship between immune cells and the risk of BC. For significant outcomes, we performed reverse MR analysis to address potential reverse causality. In the exposure-outcome analysis, we used MR with at least 2 SNPs as IV. Five MR methods were used to estimate causality: MR-Egger, weighted median, inverse variance weighted (IVW), simple mode, and weighted mode. These are widely recognized for their robustness in causal reasoning, and were chosen as the primary method for estimating causal effects. The IVW approach was used to estimate causality in the exposure-outcome analysis.^[9]^

#### 2.3.3. Assessment of heterogeneity and horizontal polytropy

To maintain the validity of the independence and exclusivity assumptions, it was important to ensure that IV did not influence the outcome through factors unrelated to the exposure variable. Horizontal pleiotropy was assessed using the MR Egger intercept test, and a *P* > .05 indicated that horizontal pleiotropy did not exist.^[7]^ Cochran *Q*-test was used to test for heterogeneity of the MR results, and a *P* > .05 indicated that heterogeneity did not exist.^[10]^ Sensitivity analyses were conducted by the “leave-one-out” method to assess whether the causal relationship between exposure and outcome influenced by any single SNP.^[11]^ All the above MR analysis procedures were performed using the “TwoSampleMR” and “gwasglue” methods in R version 4.4.1 (R Core Team; http://www.Rproject.org).

#### 2.3.4. Overall causal effect analysis

To comprehensively investigate the causal relationship between immune cell phenotypes and BC, we conducted two-way, two-sample Mendelian randomization (MR) analyses involving 731 immune cell phenotypes and their associations with BC. Initially, the causal relationships were assessed using the IVW method through the “MendelianRandomization” R package, without accounting for heterogeneity or horizontal pleiotropy.^[12]^ To enhance the robustness and reliability of the results, the MR-Egger regression and weighted median methods were subsequently applied for validation.^[13,14]^

#### 2.3.5. Mediated effects analysis

Mediation analyses were performed to explore whether metabolites mediate a causal pathway from immune cells to BC outcomes using a two-step MR approach. The overall effect of immune cells on BC can be decomposed into a direct effect of the immune cell phenotype on BC, and an indirect effect mediated by intermediates.^[15]^ The overall impact of immune traits on BC can be divided into direct and indirect effects. Indirect effects were calculated as the product of the causal relationship (*Beta1*) between immune cells and metabolites as well as the causal relationship (*Beta2*) between metabolites and BC.^[16]^ The mediation ratio was calculated as the mediation effect divided by total effect^[17]^:


$$\begin{array}{l} R=\frac{Beta\;\;\;1蜧Beta\;\;\;2}{Beta\;\;\;0} \end{array}$$

The direct effects (*Beta3*) were equal to the total effect (*Beta0*) minus the indirect effects (Beta1*Beta2)^[18]^:


$$\begin{array}{l} Beta\;3=Beta\;0-Beta\;1蜧Beta2 \end{array}$$

## 3. Results

### 3.1. Genetic causality between immune cells and BC

**Figure 2.**
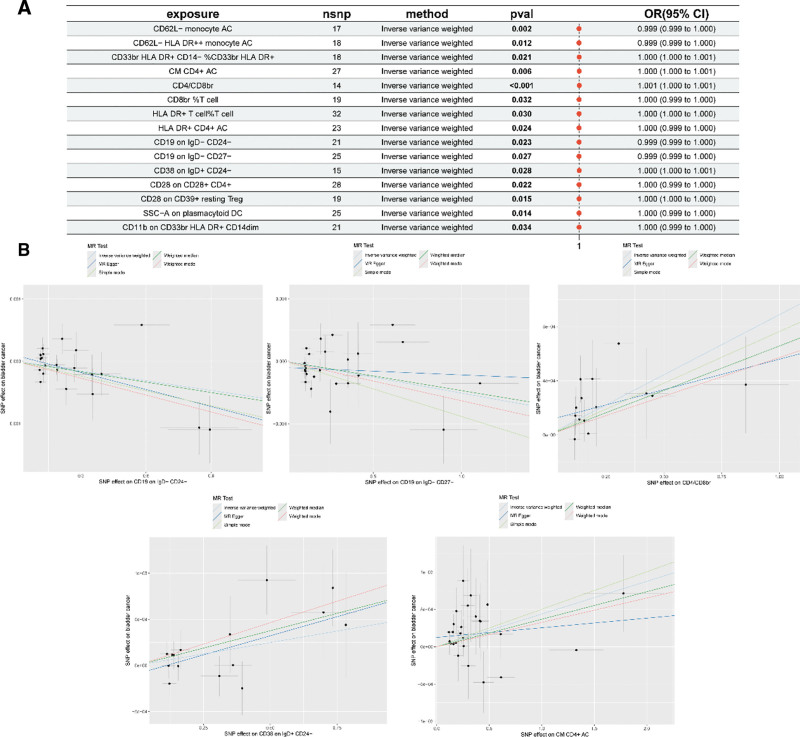
(A) Forest plot showing the causal relationships between 15 immune cell types and bladder cancer. (B) Scatter plots for 5 representative immune cell phenotypes, illustrating the effect estimates and standard errors of each SNP on the immune phenotype (*X*-axis) and bladder cancer (*Y*-axis). Each point represents an instrumental variable (IV), and the plot includes fitted lines from various Mendelian randomization methods, including inverse-variance weighted (IVW), MR-Egger, weighted median, and simple mode, to assess the robustness of the causal estimates. CD19⁺IgD⁻CD24⁻ and CD19⁺IgD⁻CD27⁻ cells were found to be associated with a reduced risk of bladder cancer, whereas CD4/CD8^bright^, CD38⁺IgD⁺CD24⁻, and central memory (CM) CD4⁺ activated cells (AC) were associated with an increased risk. CI = confidence interval, OR = odds ratio, SNP = single nucleotide polymorphism.

The correlation analyses were performed to eliminate chain imbalances and weak IVs, resulting in the identification of 18,621 SNPs associated with immune cells with a minimum *F*-value of 19.53 using quantitative tools. To investigate the overall causal effect of immune cells on BC, we performed two-sample Mendelian randomization (TSMR) analyses using 5 methods: IVW, weighted median, simple mode, weighted mode, and MR-Egger. Subsequently, we used the threshold *P* < .05 derived from the IVW method to detect 16 immune cells causally associated with BC, including T cells (CM CD4+ AC, CD4/CD8br, CD8br %T cell, HLA DR+ T cell %T cell, HLA DR+ CD4+ AC, CD19 on IgD− CD24−, CD25 on CD39+ resting regulatory T [Treg]), B cells (CD19 on IgD− CD27−, CD38 on IgD+ CD24−, CD28 on CD28+ CD4+, CD28 on CD39+ resting Treg), monocyte cells (CD62L− monocyte AC, CD62L− HLA DR++ monocyte AC), myeloid cells (CD33br HLA DR+ CD14− %CD33br HLA DR+, CD11b on CD33br HLA DR+ CD14dim), plasmacytoid dendritic cells (pDC) (SSC-A on plasmacytoid DC). In this study, we performed reverse MR analysis with BC as an exposure factor and 16 immune cell phenotypes as outcome factors. Our findings showed no reverse causality (*P* > .05) between BC and the 15 immune cell phenotypes. However, reverse causality was observed for one immune cell phenotype (*P* < .05); therefore, CD25 on CD39+ resting Tregs was excluded. In addition, 11 immune cell phenotypes were negatively associated with BC, and 4 were positively associated. Meanwhile, the test of multiplicity and heterogeneity yielded results (*P* > .05) with a consistent direction of ORs, and leave-one-out sensitivity analysis confirmed the robustness of the MR results (Fig. 2, Table 1).

**Table 1:** Mendelian randomization analysis of immune cells and bladder cancer.

Exposure	Method	Nsnp	Beta	SE	*P*-val	Pleiotropy	Heterogeneity	RevPvale
CD62L− monocyte AC	IVW	17	−0.000705457	0.000232857	.002449007	0.904514294	0.45108964	0.264197877
CD62L− HLA DR++ monocyte AC	IVW	18	−0.000663598	0.000263556	.011806743	0.64212189	0.54134284	0.449976501
CD33br HLA DR+ CD14− %CD33br HLA DR+	IVW	18	0.000324511	0.000140503	.020908324	0.089434862	0.592847871	0.77823041
CM CD4+ AC	IVW	27	0.000438861	0.000159215	.005843978	0.07376658	0.831651648	0.212331087
CD4/CD8br	IVW	14	0.000887721	0.000248336	.00035067	0.239541989	0.796021902	0.989409668
CD8br %T cell	IVW	19	−0.000448204	0.000209451	.032363103	0.084345437	0.244448036	0.723923349
HLA DR+ T cell%T cell	IVW	32	−0.000247053	0.000113832	.029981463	0.138489033	0.361865651	0.716265763
HLA DR+CD4+ AC	IVW	23	−0.000487661	0.000216299	.024160599	0.276157626	0.070483553	0.291022594
CD19 on IgD− CD24−	IVW	21	−0.000520096	0.000229416	.023387427	0.261092829	0.217340761	0.374596636
CD19 on IgD− CD27−	IVW	25	−0.000508478	0.000230027	.027069552	0.212012777	0.126478444	0.437236491
CD38 on IgD+ CD24−	IVW	15	0.000496815	0.00022601	.027935194	0.310776526	0.493769101	0.687729526
CD28 on CD28+ CD4+	IVW	28	−0.00029823	0.000129736	.021519618	0.702878681	0.784913233	0.051456115
CD28 on CD39+ resting Treg	IVW	19	−0.000250477	0.000103235	.015253905	0.393224747	0.237972715	0.657141481
SSC-A on plasmacytoid DC	IVW	25	−0.00037218	0.000150992	.013705485	0.306308088	0.822970804	0.238302703
CD11b on CD33br HLA DR+ CD14dim	IVW	21	−0.00030241	0.000143023	.034479349	0.629497968	0.868123394	0.646727819

AC = activated cells, IVW = inverse variance weighted.

### 3.2. Genetic causality of metabolites and BC

**Figure 3.**
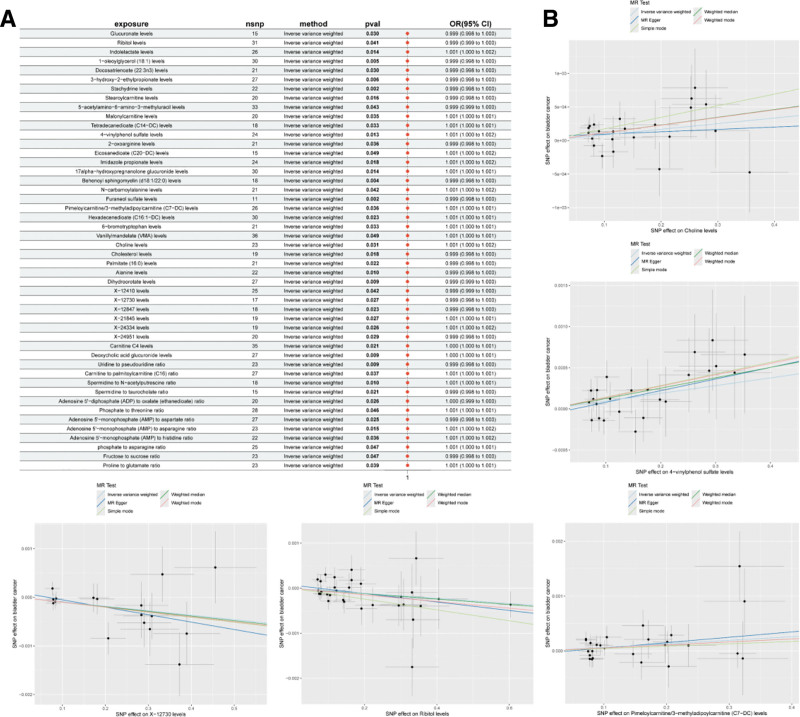
(A) Forest plot of 48 plasma metabolites causally associated with bladder cancer. (B) Scatter plots for 5 representative metabolites, illustrating the effect estimates and standard errors of each SNP on metabolite levels (*X*-axis) and bladder cancer (*Y*-axis). Each point represents an instrumental variable (IV), and the plots include fitted lines from multiple Mendelian randomization methods, including inverse-variance weighted (IVW), MR-Egger, weighted median, and simple mode, to assess the robustness of the causal estimates. Among these metabolites, X-12730 and ribitol levels were associated with a reduced risk of bladder cancer, whereas choline levels, 4-vinylphenol sulfate levels, and pimeloylcarnitine/3-methyladipoylcarnitine (C7-DC) levels were associated with an increased risk. CI = confidence interval, OR = odds ratio, SNP = single nucleotide polymorphism.

By selecting IVs, we performed an association analysis to eliminate chain imbalances and weak IVs, which led to the identification of 34,843 SNPs associated with plasma metabolites, with a minimum *F*-statistic of 19.50. Using the same research approach, we initially identified 49 plasma metabolites with potential causal relationships with BC. Due to significant heterogeneity observed in the analysis of 2-hydroxysebacate (*P* < .05), it was excluded from further analysis. Among the remaining 48 metabolites included in the analysis, 42 were identified metabolites and 6 were unknown compounds. Among the known metabolites, 22 may be associated with an increased risk of BC, including choline, pimeloylcarnitine/3-methyladipoylcarnitine (C7-DC), and 4-vinylphenol sulfate (4-VPS) levels. In contrast, 20 metabolites were potentially negatively associated with BC risk, including ribitol, 1-oleoylglycerol (18:1), and glucuronate levels (Fig. 3, Table 2).

**Table 2:** Mendelian randomization analysis of metabolites and bladder cancer.

Exposure	Method	Nsnp	Beta	SE	*P*-val	Pleiotropy	Heterogeneity
Glucuronate levels	IVW	15	−0.001073114	0.000493748	.029749906	0.672252265	0.508686618
Ribitol levels	IVW	31	−0.000564724	0.000276122	.040835359	0.322705789	0.510022801
Indolelactate levels	IVW	26	0.000863361	0.000351756	.014110884	0.896340896	0.492778623
1-Oleoylglycerol (18:1) levels	IVW	30	−0.000920088	0.000326152	.004786855	0.708129301	0.99698244
Docosatrienoate (22:3n3) levels	IVW	21	−0.000810321	0.000373952	.030241769	0.962491013	0.123548475
3-Hydroxy-2-ethylpropionate levels	IVW	27	−0.000979664	0.000355352	.005835483	0.896340896	0.351617201
Stachydrine levels	IVW	22	−0.001232526	0.00039843	.001978389	0.2604085	0.685471992
Stearoylcarnitine levels	IVW	20	−0.000848802	0.000352243	.015965453	0.562548286	0.562160925
5-Acetylamino-6-amino-3-methyluracil levels	IVW	33	−0.000512983	0.00025364	.0431262	0.67044135	0.493700068
Malonylcarnitine levels	IVW	20	0.000771063	0.000365568	.034925568	0.987882437	0.470040683
Tetradecanedioate (C14-DC) levels	IVW	18	0.000580039	0.000271679	.032759906	0.756005857	0.619121414
4-Vinylphenol sulfate levels	IVW	24	0.000941283	0.000378089	.012789434	0.542426062	0.840201128
2-Oxoarginine levels	IVW	21	−0.000830219	0.000396509	.036275909	0.273105846	0.459863594
Eicosanedioate (C20-DC) levels	IVW	15	0.000860592	0.000437744	.049302087	0.792420863	0.775798229
Imidazole propionate levels	IVW	24	0.000981042	0.000415176	.018129807	0.721914496	0.142885881
17-alpha-hydroxypregnanolone glucuronide levels	IVW	30	0.000640777	0.000261465	.014257319	0.42036626	0.850243988
Behenoyl sphingomyelin (d18:1/22:0) levels	IVW	18	−0.001323989	0.000454063	.00354697	0.773036829	0.672919237
N-carbamoylalanine levels	IVW	21	0.000821954	0.000404294	.042046619	0.163320963	0.495662387
Furaneol sulfate levels	IVW	11	−0.001194722	0.000387159	.002029593	0.758521985	0.387838131
Pimeloylcarnitine/3-methyladipoylcarnitine (C7-DC) levels	IVW	26	0.000644744	0.000307827	.036215529	0.573274801	0.63601432
Hexadecenedioate (C16:1-DC) levels	IVW	30	0.000535537	0.000235754	.023111081	0.950517512	0.159826409
6-Bromotryptophan levels	IVW	21	0.000564317	0.000264941	.033173746	0.802329031	0.617427275
Vanillylmandelate (VMA) levels	IVW	36	0.000651841	0.000330953	.048885335	0.852803281	0.328929123
Choline levels	IVW	23	0.000837533	0.000387371	.030610621	0.577209394	0.829305676
Cholesterol levels	IVW	19	−0.001030859	0.000435026	.01780495	0.659744227	0.902878224
Palmitate (16:0) levels	IVW	21	−0.000940653	0.000412139	.022467497	0.430423177	0.749625121
Alanine levels	IVW	22	−0.00102685	0.000397194	.009730642	0.58687087	0.316597781
Dihydroorotate levels	IVW	27	−0.00069695	0.000268615	.009469867	0.453403803	0.535758665
X-12410 levels	IVW	25	−0.000629179	0.000308666	.041511874	0.427454548	0.780602082
X-12730 levels	IVW	17	−0.000917951	0.000414738	.02687506	0.342444621	0.494976624
X-12847 levels	IVW	18	−0.000916559	0.00040313	.022989764	0.914878806	0.971327996
X-21845 levels	IVW	19	0.000740044	0.000334858	.027103601	0.447927278	0.572496689
X-24334 levels	IVW	19	0.000835775	0.000375051	.025851911	0.102874872	0.801032771
X-24951 levels	IVW	20	−0.000960857	0.000439041	.028630347	0.585691878	0.588387057
Carnitine C4 levels	IVW	35	0.000389928	0.000168303	.020513431	0.123832776	0.387945445
Deoxycholic acid glucuronide levels	IVW	27	0.000458834	0.000176529	.009344124	0.479610339	0.879728137
Uridine to pseudouridine ratio	IVW	23	−0.00100743	0.000383588	.008631073	0.802356942	0.812061777
Carnitine to palmitoylcarnitine (C16) ratio	IVW	27	0.000763943	0.000366825	.037289566	0.098773213	0.230796097
Spermidine to N-acetylputrescine ratio	IVW	18	0.000805473	0.000313881	.010282831	0.262554894	0.500339034
Spermidine to taurocholate ratio	IVW	15	−0.001013176	0.000438562	.02087584	0.992689842	0.425962635
Adenosine 5′-diphosphate (ADP) to oxalate (ethanedioate) ratio	IVW	20	−0.00043365	0.00019485	.026043769	0.674464959	0.275077181
Phosphate to threonine ratio	IVW	28	0.000687417	0.000343907	.045624904	0.653124394	0.630672608
Adenosine 5′-monophosphate (AMP) to aspartate ratio	IVW	27	−0.000895149	0.000398034	.024517199	0.646932116	0.203770025
Adenosine 5′-monophosphate (AMP) to asparagine ratio	IVW	23	0.000882091	0.000362747	.015028189	0.221894762	0.546281333
Adenosine 5′-monophosphate (AMP) to histidine ratio	IVW	22	0.00084553	0.000403867	.036297128	0.161108246	0.813517111
phosphate to asparagine ratio	IVW	25	0.000517017	0.000260738	.04737847	0.469564479	0.461583735
Fructose to sucrose ratio	IVW	23	−0.000840704	0.000423094	.046917858	0.683740135	0.218612378
Proline to glutamate ratio	IVW	23	0.000767642	0.000371864	.038988355	0.672364772	0.50566083

IVW = inverse variance weighted.

### 3.3. Mediated Mendelian randomization analysis

**Figure 4.**
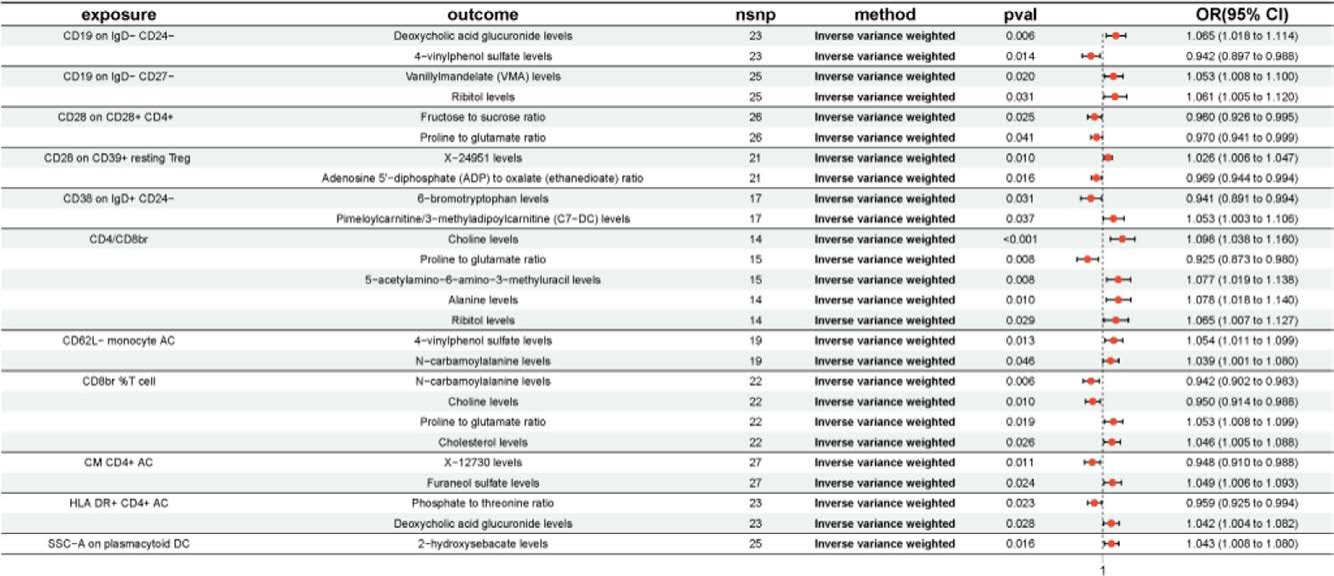
Forest diagram of immune cells with metabolites. CI = confidence interval, OR = odds ratio, SNP = single nucleotide polymorphism.

Based on the previously identified immune cells and plasma metabolites, mediation was further calculated using the TSMR method. We performed MR analysis of the immune cell phenotype in response to plasma metabolites using 15 selected immune cells as exposure factors and 48 plasma metabolites as outcome indicators, we performed an MR analysis of the immune cell phenotype in response to plasma metabolites. This analysis revealed causal relationships between the 11 immune cell phenotypes and 19 plasma metabolites, yielding an effect size of 1 from the immune cell phenotype to a metabolite. Our study found a positive correlation between CD4/CD8br and choline levels, a negative correlation between CD19 on IgD− CD24− and 4-VPS levels, and a CD19 on IgD− CD27− and ribitol levels there was a positive correlation, among other findings. Further analyses suggest that individual immune cell phenotypes may be causally linked to multiple metabolites. For example, CD4/CD8br was positively correlated with choline levels, but negatively correlated with the proline-to-glutamate ratio. Similarly, CD28 in CD28+ CD4+ cell showed a negative correlation with the fructose-to-sucrose ratio while a negative correlation with the proline-to-glutamate ratio. In addition, CD8br %T cell were negatively correlated with N-carbamoylalanine and choline levels, but positively correlated with the proline to glutamate ratio and cholesterol levels. When 19 plasma metabolites were considered as exposure factors and BC as an outcome, MR analysis and MR-PRESSO test were performed (*P* > .05), which showed no multiplicity of effects and unbiased SNPs. The effect sizes of the metabolites on BC were determined to be 2, and the overall effect of immune cells on BC was subsequently calculated (Fig. 4, Table 3, Table S1, Supplemental Digital Content, https://links.lww.com/MD/Q694).

**Table 3:** Mendelian randomization analysis of immune cells and metabolites.

Exposure	Outcome	Method	Nsnp	Beta	SE	*P*val	Pleiotropy	Heterogeneity
CD62L− monocyte AC	4-Vinylphenol sulfate levels	IVW	19	0.052975	0.021327	.012996	0.54723	0.156234
CD62L− monocyte AC	N-Carbamoylalanine levels	IVW	19	0.038573	0.019375	.04649	0.675362	0.94402
CM CD4+ AC	Furaneol sulfate levels	IVW	27	0.047458	0.021097	.024479	0.33262	0.731649
CM CD4+ AC	X-12730 levels	IVW	27	−0.05298	0.020882	.011181	0.162045	0.241126
CD4/CD8br	Ribitol levels	IVW	14	0.063061	0.028827	.028699	0.411288	0.740109
CD4/CD8br	5-Acetylamino-6-amino-3-methyluracil levels	IVW	15	0.074318	0.028212	.008432	0.728498	0.602304
CD4/CD8br	Choline levels	IVW	14	0.093112	0.028287	.000996	0.112575	0.39616
CD4/CD8br	Alanine levels	IVW	14	0.074704	0.028811	.009518	0.925977	0.947555
CD4/CD8br	Proline to glutamate ratio	IVW	15	−0.07821	0.029361	.007724	0.537947	0.280965
CD8br %T cell	N-carbamoylalanine levels	IVW	22	−0.06001	0.021912	.006165	0.373647	0.404012
CD8br %T cell	Choline levels	IVW	22	−0.05082	0.01972	.00997	0.076184	0.678741
CD8br %T cell	Cholesterol levels	IVW	22	0.044784	0.02008	.025733	0.689419	0.902292
CD8br %T cell	Proline to glutamate ratio	IVW	22	0.05163	0.02204	.019149	0.753992	0.248405
HLA DR+ CD4+ AC	Deoxycholic acid glucuronide levels	IVW	23	0.041456	0.018894	.028222	0.696276	0.168556
HLA DR+ CD4+ AC	Phosphate to threonine ratio	IVW	23	−0.04212	0.01856	.023237	0.949595	0.18766
CD19 on IgD− CD24−	4-Vinylphenol sulfate levels	IVW	23	−0.06027	0.024512	.013938	0.393824	0.332163
CD19 on IgD− CD24−	Deoxycholic acid glucuronide levels	IVW	23	0.063011	0.023134	.006454	0.960251	0.937835
CD19 on IgD− CD27−	Ribitol levels	IVW	25	0.059288	0.027523	.031228	0.273614	0.212937
CD19 on IgD− CD27−	Vanillylmandelate (VMA) levels	IVW	25	0.051717	0.022296	.020364	0.67025	0.406206
CD38 on IgD+ CD24−	Pimeloylcarnitine/3-methyladipoylcarnitine (C7-DC) levels	IVW	17	0.051875	0.024811	.036545	0.789512	0.797948
CD38 on IgD+ CD24−	6-Bromotryptophan levels	IVW	17	−0.06053	0.027981	.030521	0.210795	0.210313
CD28 on CD28+ CD4+	Fructose to sucrose ratio	IVW	26	−0.04119	0.018341	.024727	0.335881	0.122878
CD28 on CD28+ CD4+	Proline to glutamate ratio	IVW	26	−0.0309	0.01512	.040986	0.917296	0.719477
CD28 on CD39+ resting Treg	X-24951 levels	IVW	21	0.025977	0.010146	.010462	0.187173	0.340688
CD28 on CD39+ resting Treg	Adenosine 5′-diphosphate (ADP) to oxalate (ethanedioate) ratio	IVW	21	−0.03186	0.013204	.015835	0.945799	0.772005
SSC-A on plasmacytoid DC	2-Hydroxysebacate levels	IVW	25	0.042265	0.017585	.016243	0.586718	0.498197

AC = activated cells, IVW = inverse variance weighted.

### 3.4. Analysis of intermediation effects

**Figure 5.**
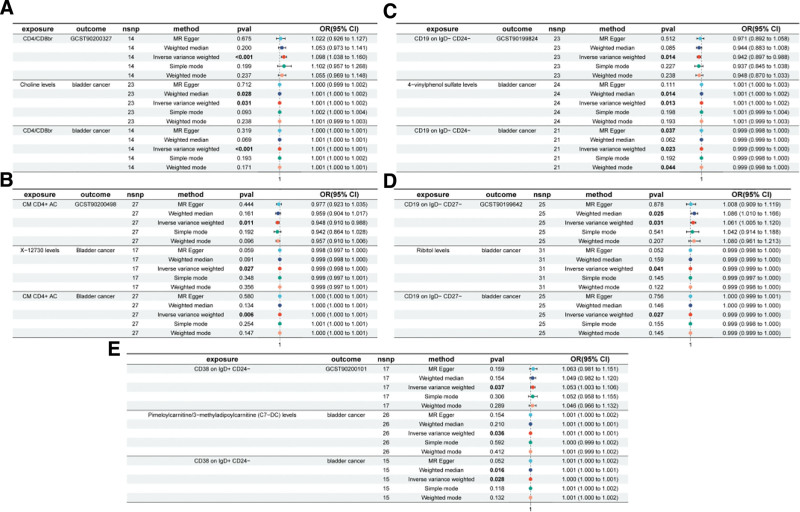
Forest plot of 5 immune cell phenotypes, 5 plasma metabolites, and bladder cancer. CI = confidence interval, OR = odds ratio, SNP = single nucleotide polymorphism.

Ultimately, we performed mediation analyses to elucidate the causal relationship between immune cell phenotypes and BC mediated by plasma metabolites (Table 4). Five plasma metabolites were found to mediate the relationship between the 5 immune cell phenotypes and BC, 4 of which were known metabolites, one of which remains to be identified. These results revealed a causal relationship between CM CD4+ AC on T cells and BC, which was mediated by the levels of the unknown metabolite X-12730, with the highest percentage of mediation at 11.1% (Fig. 5A). The causal relationship between CD4/CD8br in T cells and BC was mediated by choline levels, with a mediation percentage of 8.78% (Fig. 5B). There was a causal relationship between CD19 on IgD− CD24− on B cells and BC, mediated by 4-VPS levels, with a mediation ratio of 10.9% (Fig. 5C). There was a causal relationship between CD19 on IgD− CD27− on B cells and BC, mediated by ribitol levels of 6.58% (Fig. 5D). There was a causal association between CD38 on IgD+ CD24− in B cells and BC, mediated by pimeloylcarnitine/3-methyladipoylcarnitine (C7-DC) levels, mediated by 6.73% (Fig. 5C) of 6.73% (Fig. 5E).

**Table 4:** Mendelian randomization analysis of causal relationship between immune cells, plasma metabolites and bladder cancer.

Immune cell	Metabolite	Outcome	Mediated effect	Mediated proportion
CD4/CD8br	Choline levels	Bladder cancer	7.8e−05 (−0.00508, 0.00524)	8.78% (−573%, 590%)
CM CD4+ AC	X-12730 levels	Bladder cancer	4.86e−05 (−0.00212, 0.00222)	11.1% (−483%, 505%)
CD19 on IgD− CD24−	4-Vinylphenol sulfate levels	Bladder cancer	−5.67e−05 (−0.00295, 0.00284)	10.9% (568%, −546%)
CD19 on IgD− CD27−	Ribitol levels	Bladder cancer	−3.35e−05 (−0.00323, 0.00316)	6.58% (636%, −622%)
CD38 on IgD+ CD24−	Pimeloylcarnitine/3-methyladipoylcarnitine (C7-DC) levels	Bladder cancer	3.34e−05 (−0.00249, 0.00256)	6.73% (−501%, 515%)

AC = activated cells.

## 4. Discussion

The MR study demonstrated a causal relationship between the 16 immune cell phenotypes and BC. However, reverse MR analysis showed that 15 immune cell phenotypes were not causally associated with BC, while 1 does showed a causal relationship. Further mediation analyses using TSMR and multivariable Mendelian randomization showed that the 5 cellular phenotypes could be causally linked to BC via 5 plasma metabolites (including one unidentified metabolite), with the highest percentage of mediation (11.1%) for the X-12730 levels (unknown metabolite).

This study confirmed causal relationships between 16 immune cell phenotypes and BC (Table 1), with one phenotype subsequently excluded. In the tumor microenvironment of BC, CD25^+^ CD39^+^ resting Tregs are markedly increased. Through the activity of CD39 and tumor-upregulated CD73, these cells synergistically promote adenosine production, thereby suppressing the function of effector T cells and NK cells, contributing to the establishment of an immunosuppressive environment and facilitating tumor immune evasion.^[19]^ Research has shown that Treg levels are elevated in patients with early-stage BC (pT1–pT2), while in advanced stages (pT3–pT4), Treg levels decline, suggesting that Tregs play a more prominent immunosuppressive role during early tumor development.^[20]^ Our reverse MR analysis further demonstrated that BC may influence the levels of CD25^+^ CD39^+^ resting Tregs (*P* < .05), indicating that this immune phenotype may be regulated by the tumor itself. Therefore, this phenotype was excluded from the forward MR analysis to avoid confounding the direction of causality.

Among the 15 immune cell phenotypes with causal relationships identified through MR analysis, only a subset (CD4/CD8br on T cells, CM CD4^+^ AC, CD19 on IgD^−^ CD24^−^, CD19 on IgD^−^ CD27^−^, and CD38 on IgD^+^ CD24^−^ on B cells) are likely to contribute to BC development through blood-derived metabolic mediators. Treg cells, a subpopulation of T cells specialized for immunosuppression, play a key role in maintaining immune self-tolerance and homeostasis. Depletion of Treg cells in tumor immunity, or attenuation of immunosuppression by modulating Treg cells, can stimulate or enhance the body’s immune response against tumors. This finding advances the development of cancer immunotherapy strategies targeting Treg cells. A previous study found a positive correlation between Treg cells and BC. Treg levels in MIBC constitute an independent predictor of prognosis in BC and are an important player in the TME. The imbalance of Treg cells during BC progression and the regulation of Treg cells to ameliorate BC,^[21]^ this emphasizes the importance of T cells in BC. Previous studies have shown a correlation between T cell changes and BC. Our study elaborates in depth that there is a causal relationship between T cell and BC, and shows in more detail that T cell subtypes play an important role in the physiological activities of BC, which provides new perspectives for further research on the role of T cells in the diagnosis and treatment of BC. B cells are important contributors to the antitumor immune response by presenting tumor antigens to T cells.^[22]^ Liu et al showed that the function of B cells varies across different types of cancers.^[23]^ B cells can be activated by tumor cells, which secrete immunoglobulins to inhibit tumor growth.^[24]^ Conversely, B-cell infiltration also promotes tumor invasion and metastasis.^[25]^ Kroeger et al indicated that in BC patients, there is an increase in clonal B cell expansion and plasmablast infiltration within tumor tissues. The above studies suggest that B cell activation is associated with immune-mediated mechanisms in the pathogenesis of BC; however, the causality of the link between B cells and BC has not been clarified. A causal relationship between specific B-cell phenotypes and BC was found in this study, in which an increase in B cells was associated with BC progression.^[25]^ This is consistent with our findings that CD38 on B cells increases the risk of BC. In addition, this study found a causal relationship between CD19 and reduced risk in BC patients; however, further studies are needed to confirm this result. As innate immune cells with a high degree of plasticity, monocytes exhibit significant diversity in maintaining physiological homeostasis, responding to inflammatory responses, tumor formation, and tumor-induced changes in the systemic and local microenvironments, which affect monocyte characteristics, differentiation, and distribution. In addition, monocytes and their associated subpopulations play a critical role in regulating tumor growth and metastasis in the development of multiple cancers.^[26]^ CD62L− monocyte AC, CD62L− HLA DR++ monocyte AC are different monocyte phenotypes of monocytes. Cheah et al found that monocytes promote the formation of the tumor microenvironment by releasing inflammatory factors, such as IL-6 and IL-8. Monocytes play a critical role in regulating immune responses and promoting tumor growth.^[27]^ Monocytes are also etiologically associated with BC.^[28]^ Monocytes and their associated markers play an important role in the development and prognosis of BC, providing new perspectives for the diagnosis and treatment of BC.^[29,30]^ Based on previous studies, we elaborated on the causal relationship between monocytes and BC in greater depth, and showed in greater detail that monocytes play an important role in the physiological activities of BC. This provides new perspectives for further research on the role of monocytes in the diagnosis and treatment of BC. Bone marrow cells constitute a key cellular component of immune cells that infiltrate tumors and play an important role in the regulation of tumor inflammation and angiogenesis.^[31]^ Eruslanov et al indicated that peripheral blood from BC patients contains 2 major CD11b myeloid cell subsets: granulocyte-type CD15-high CD33-low cells and monocyte-type CD15-low CD33-high cells. Highly active inflammatory myeloid cells serve as a major source of various chemokines and cytokines, which may trigger inflammatory responses and immune dysfunction in BC.^[32]^ Based on previous studies, we showed in greater detail that myeloid cell subtypes play an important role in the physiological activities of BC, providing new perspectives for further research on the role of myeloid cells in the diagnosis and treatment of BC. pDC are bone marrow-derived immune cells that have the ability to express large amounts of type I and type III interferon and to differentiate into antigen-presenting dendritic cells as a result of stimulation with pathogen-derived nucleic acids.^[33]^ As multifunctional bone marrow-derived immune cells, pDCs play a crucial role in bridging innate and adaptive immune systems. The activation of pDC by Toll-like receptor agonists has been shown to be effective in the treatment of certain oncologic diseases.^[31]^ We showed in greater depth that dendritic cells play an important role in the physiological activities of BC, providing new perspectives for further investigation of the role of dendritic cells in the diagnosis and treatment of BC.

Metabolites, as a means of intercellular communication, not only play a role in cancer growth, but also play a crucial role in the early identification of at-risk individuals and disease prevention.^[34]^ Kouznetsova et al identified key metabolites and genes associated with BC stages, providing a foundation for the development of more robust analytical tools to discover novel biomarkers and targeted therapeutic compounds. Their findings also demonstrated potential applications in disease diagnosis and monitoring.^[35]^ Various immune cells mediates the critical roles of multiple metabolites in diseases such as pancreatic cancer, colorectal cancer, and chronic obstructive pulmonary disease.^[18,36,37]^ Through Mendelian randomization analysis, we identified 48 metabolites associated with BC. Among these, only 5 metabolites were involved in mediating regulation: choline levels, 4-VPS levels, X-12730 levels, ribitol levels, and pimeloylcarnitine/3-methyladipoylcarnitine (C7-DC) levels. Choline is a water-soluble quaternary amine that is commonly grouped with vitamin B owing to its chemical similarity, and is a key nutrient in humans, which has a variety of key functions in the human body, particularly in neurochemical processes.^[38]^ Studies over the past decade have shown that choline metabolism undergoes significant changes in a variety of cancers, with increased levels of phosphorylcholine and total choline recognized as potential endogenous biomarkers, and the associated enzymes and metabolic pathways may be new targets for anticancer therapy.^[39]^ Ossoliński et al found that choline had a significant diagnostic enhancement and served as an important discriminator for distinguishing cancerous features from healthy phenotypes in BC patients.^[40]^ 4-VPS levels are a key metabolite of 4-vinylphenol, a xenobiotic generated from styrene metabolism.^[41]^ Chen et al found in a 2-sample Mendelian randomization study of plasma metabolites and 7 cancers that 4-VPS was significantly negatively associated with cancer risk.^[42]^ Zoledronic acid is primarily used to treat cancer-related bone complications, and Li et al.‘s findings suggest that 4-VPS may be associated with response to zoledronic acid treatment.^[41]^ 4-VPS showed the most pronounced protective effect against prostate cancer and renal cell carcinoma.^[43]^ Ribitol is a metabolite in nature and has been tested in animal models and human clinical trials without serious side effects.^[44]^ Tucker et al indicated that metabolic reprogramming in breast cancer cells revealed the differential effects of ribitol on cellular metabolism and gene expression, providing new insights into the development of cancer-targeted therapies targeting metabolic pathways.^[45]^ pimeloylcarnitine/3-methyladi¬poylcarnitine (C7-DC) is a metabolite of acylcarnitines. Methyladipoylcarnitine (C7-DC) is an acylcarnitine metabolite belonging to a family of nonprotein amino acids. They play an important role in the metabolism of long-chain fatty acids, acting as carriers to transport activated long-chain fatty acids to the mitochondria for β-oxidation and to provide energy for cellular functions.^[46]^ Huo et al found that high serum levels of decanoylcarnitine, heptadecanoylcarnitine, and tetradecadienylcarnitine significantly reduced the risk of Alzheimer disease, and that this predictive effect was independent of age, sex, and educational background.^[47]^ In addition, one of the mediators identified in this study, X-12730, remains an unknown metabolite that has not yet been annotated in public databases. In the future, its potential biological functions could be inferred through structure prediction based on MS/MS data and pathway enrichment analyses. Targeted metabolomics detection of X-12730 and functional validation in BC cohorts may further elucidate its potential role in tumorigenesis.In summary, we believe these metabolites not only hold significant biological relevance but also possess the potential to be translated into early detection biomarkers or therapeutic targets. Future research could advance their clinical utility through multicenter clinical validation, in vivo functional studies, and targeted metabolomic investigations.

This study found a negative causal relationship between the CD19⁺ IgD⁻ CD24⁻ B cell subset and BC risk, with the effect mediated by 4-VPS levels. CD19 is a key molecule involved in B cell activation and signal transduction; its reduced expression may indicate functional impairment of antitumor B cell subsets.^[48]^ 4-VPS is a known aromatic hydrocarbon metabolite derived from tobacco smoke and industrial pollutants. Current evidence linking it to cancer or inflammatory conditions primarily stems from statistical associations observed in epidemiological and genetic studies, such as Mendelian randomization. To date, no studies have elucidated its biological functions in these pathological processes at the mechanistic or experimental level. Our results suggest that CD19 expression may indirectly influence BC risk by regulating the metabolic clearance of 4-VPS. In addition, the causal relationship between the CD19⁺ IgD⁻ CD27⁻ B cell subset and BC risk was mediated by ribitol levels. Ribitol, a pentitol metabolite, is closely related to the cellular redox state and energy metabolism. Previous studies have suggested that ribitol may regulate apoptosis and antioxidant processes, thereby influencing tumor progression.^[45,49]^ We observed a positive correlation between CD19 expression and ribitol levels, and a negative correlation between ribitol levels and BC risk, indicating that this B cell subset may exert protective effects through the enhancement of ribitol-related antitumor metabolic pathways. The CD38⁺ IgD⁺ CD24⁻ B cell subset was positively associated with BC risk, with the effect mediated by the acylcarnitine metabolite pimeloylcarnitine/3-methyladipoylcarnitine (C7-DC). CD38 is a NAD⁺-consuming enzyme that is upregulated in various tumor immunosuppressive environments, facilitating immune evasion.^[50]^ Meanwhile, C7-DC is involved in mitochondrial fatty acid β-oxidation, and its elevation may reflect a state of metabolic reprogramming that supports the energy demands and biosynthetic precursors required by rapidly proliferating tumor cells.^[51]^ Our findings suggest that CD38-high B cells may promote tumor-associated metabolic adaptation through regulation of lipid metabolism. We also identified a positive causal relationship between the CD4/CD8br T cell subset (characterized by an elevated CD4⁺/CD8⁺ ratio) and BC, with choline levels mediating this effect. Choline is a key intermediate in phospholipid metabolism of cell membranes and plays an important role in tumor cell proliferation and signaling. Elevated choline metabolism has been observed in various malignancies and is associated with poor prognosis.^[52,53]^ An increased CD4/CD8 ratio typically reflects immune imbalance, particularly reduced activity of cytotoxic CD8⁺ T cells, which may weaken antitumor immunity.^[54]^ Therefore, we hypothesize that this T cell subset may promote BC development and progression by influencing choline metabolism. Finally, the CM CD4⁺ AC T cell subset showed a causal relationship with BC, mediated by X-12730 levels. CM CD4⁺ AC was positively associated with BC, while it was negatively correlated with X-12730 levels, which were themselves negatively associated with BC risk. X-12730 is an unannotated metabolite. Our results confirmed the causal relationship between X-12730 levels and BC, suggesting that the CM CD4⁺ AC T cell subset may influence BC risk through this unknown metabolite. In conclusion, our study systematically revealed, from a causal inference perspective, how immune cell subtypes affect BC risk through metabolic pathways, highlighting the interplay between immune regulation and metabolic dysregulation. These findings not only enhance our understanding of the immunometabolic mechanisms underlying BC development but also provide a theoretical basis and potential targets for metabolism-mediated immunotherapeutic strategies.

### 4.1. Limitations of this study

Given the exploratory nature of this study, we did not perform multiple testing corrections such as Bonferroni or FDR for the large number of comparisons between immune phenotypes and plasma metabolites. This was intended to retain more potential causal signals for future in-depth investigation. However, this strategy may increase the risk of false positives; therefore, nominally significant findings should be interpreted with caution and validated in independent cohorts. Although we did not detect significant horizontal pleiotropy (all MR-Egger intercept *P*-values > .05), the possibility that IVs may influence outcomes through unknown biological pathways cannot be completely ruled out. Additionally, all participants in this study were of European ancestry. While this reduces population stratification bias, it also limits the generalizability of the findings to other populations. Future studies could benefit from larger sample sizes, inclusion of multi-ethnic populations, and integration of rare variant analyses to further validate and extend our findings.

## 5. Conclusions

This study comprehensively assessed the causal relationship between immune cell phenotypes, plasma metabolites, and BC. We identified 5 immune cell phenotypes that are causally associated with BC, mediated by 5 metabolites. These findings highlight the importance of the potential mechanisms between immune cells, metabolites, and BC. They may help screen people at a high risk of BC and provide insights into the early prevention and preemptive diagnosis of precancerous bladder disease.

## Acknowledgments

This is a short text to acknowledge the contributions of specific colleagues, institutions, or agencies that aided the efforts of the authors.

## Author contributions

**Conceptualization:** Lijun Wang, Zitao Zhong.

**Data curation:** Juan Yang, Zhangxiao Xu.

**Formal analysis:** Juan Yang, Zhangxiao Xu, Yuanjian Niu.

**Funding acquisition:** Lijun Wang.

**Project administration:** Bo Tao.

**Software:** Yuan Zhao, Yiran Ma.

**Validation:** Juan Yang, Zhangxiao Xu.

**Writing – original draft:** Juan Yang, Zhangxiao Xu, Bo Tao, Yunpeng He, Jian Wu.

**Writing – review & editing:** Lijun Wang, Zitao Zhong.

## Supplementary Material


