# Supplementary material for: Causal insights into immune cell profiles, plasma metabolites, and bladder cancer: A Mendelian randomization approach
Source: Medicine (Baltimore). 2025 Nov 21;104(47):e46062. doi: 10.1097/MD.0000000000046062 (PMC12643734; doi:10.1097/MD.0000000000046062)
Supplement: Supplementary file 1 [file medi-104-e46062-s001.doc]

| Supplement Table 1: F-values for the 15 immune cell phenotypes | | | | | | | | | | | | | | | | |
| --- | --- | --- | --- | --- | --- | --- | --- | --- | --- | --- | --- | --- | --- | --- | --- | --- |
| pval.exposure | chr.exposure | pos.exposure | samplesize.exposure | beta.exposure | se.exposure | id.exposure | SNP | effect_allele.exposure | other_allele.exposure | eaf.exposure | exposure | mr_keep.exposure | pval_origin.exposure | data_source.exposure | R^2^ | F |
| 1.22E-08 | 1 | 1.7E+08 | 3019 | -0.3696 | 0.0647 | ebi-a-GCST90001450 | rs4656708 | C | A | 0.0459 | CD62L- monocyte AC | TRUE | reported | igd | 0.010694 | 32.61126 |
| 3.61E-15 | 1 | 1.7E+08 | 3019 | 0.2378 | 0.03006 | ebi-a-GCST90001450 | rs4987369 | T | G | 0.2774 | CD62L- monocyte AC | TRUE | reported | igd | 0.020308 | 62.54001 |
| 3.48E-06 | 2 | 56524393 | 3019 | -0.347 | 0.07464 | ebi-a-GCST90001450 | rs62167331 | A | G | 0.0341 | CD62L- monocyte AC | TRUE | reported | igd | 0.007108 | 21.59871 |
| 4.62E-06 | 3 | 74703663 | 3019 | 0.269 | 0.05861 | ebi-a-GCST90001450 | rs13068785 | T | C | 0.0581 | CD62L- monocyte AC | TRUE | reported | igd | 0.006929 | 21.05103 |
| 5.45E-06 | 4 | 4859590 | 3019 | 0.1274 | 0.02798 | ebi-a-GCST90001450 | rs1907980 | T | G | 0.3596 | CD62L- monocyte AC | TRUE | reported | igd | 0.00682 | 20.71837 |
| 3.50E-07 | 4 | 1.36E+08 | 3019 | 0.1842 | 0.03607 | ebi-a-GCST90001450 | rs11935230 | C | G | 0.1686 | CD62L- monocyte AC | TRUE | reported | igd | 0.008564 | 26.06149 |
| 6.87E-06 | 4 | 40461712 | 3019 | 0.1235 | 0.02741 | ebi-a-GCST90001450 | rs278949 | A | G | 0.5561 | CD62L- monocyte AC | TRUE | reported | igd | 0.006679 | 20.28748 |
| 6.96E-06 | 4 | 63361327 | 3019 | -0.2033 | 0.04515 | ebi-a-GCST90001450 | rs17082777 | A | G | 0.1014 | CD62L- monocyte AC | TRUE | reported | igd | 0.006671 | 20.26149 |
| 7.66E-09 | 5 | 1.77E+08 | 3019 | -0.1525 | 0.02632 | ebi-a-GCST90001450 | rs11249570 | A | G | 0.4581 | CD62L- monocyte AC | TRUE | reported | igd | 0.010998 | 33.54904 |
| 1.05E-06 | 5 | 6308011 | 3019 | 1.724 | 0.3525 | ebi-a-GCST90001450 | rs79930588 | G | T | 0.0015 | CD62L- monocyte AC | TRUE | reported | igd | 0.007861 | 23.90388 |
| 2.75E-06 | 7 | 41362245 | 3019 | 0.859 | 0.1829 | ebi-a-GCST90001450 | rs528135834 | G | T | 0.0056 | CD62L- monocyte AC | TRUE | reported | igd | 0.007253 | 22.04302 |
| 4.37E-06 | 8 | 66059237 | 3019 | -0.1386 | 0.03012 | ebi-a-GCST90001450 | rs4484736 | A | G | 0.7173 | CD62L- monocyte AC | TRUE | reported | igd | 0.006965 | 21.16064 |
| 6.13E-07 | 9 | 1.35E+08 | 3019 | 0.1627 | 0.03256 | ebi-a-GCST90001450 | rs530377 | C | T | 0.2211 | CD62L- monocyte AC | TRUE | reported | igd | 0.008203 | 24.95276 |
| 4.12E-07 | 9 | 76145588 | 3019 | 2.05 | 0.4039 | ebi-a-GCST90001450 | rs143153359 | T | C | 0.0013 | CD62L- monocyte AC | TRUE | reported | igd | 0.008461 | 25.74377 |
| 9.30E-06 | 10 | 89954638 | 3019 | 0.352 | 0.07927 | ebi-a-GCST90001450 | rs77287889 | A | G | 0.0301 | CD62L- monocyte AC | TRUE | reported | igd | 0.006489 | 19.70515 |
| 9.33E-06 | 11 | 1.12E+08 | 3019 | 0.3974 | 0.0895 | ebi-a-GCST90001450 | rs116691483 | A | G | 0.0235 | CD62L- monocyte AC | TRUE | reported | igd | 0.006488 | 19.70252 |
| 5.12E-06 | 12 | 69744014 | 3019 | 0.222 | 0.04861 | ebi-a-GCST90001450 | rs1800973 | A | C | 0.083 | CD62L- monocyte AC | TRUE | reported | igd | 0.006861 | 20.84332 |
| 1.21E-06 | 14 | 69321561 | 3019 | 0.139 | 0.02858 | ebi-a-GCST90001450 | rs4899265 | C | A | 0.3475 | CD62L- monocyte AC | TRUE | reported | igd | 0.007774 | 23.63836 |
| 8.44E-06 | 16 | 31775655 | 3019 | 0.4639 | 0.104 | ebi-a-GCST90001450 | rs146793376 | G | T | 0.0171 | CD62L- monocyte AC | TRUE | reported | igd | 0.006547 | 19.88357 |
| 6.08E-06 | 19 | 2993427 | 3019 | -0.1417 | 0.03127 | ebi-a-GCST90001450 | rs435806 | C | T | 0.2448 | CD62L- monocyte AC | TRUE | reported | igd | 0.006756 | 20.52089 |
| 1.43E-06 | 20 | 15302488 | 3019 | 0.295 | 0.06108 | ebi-a-GCST90001450 | rs149543954 | T | G | 0.0517 | CD62L- monocyte AC | TRUE | reported | igd | 0.007667 | 23.31085 |
| 5.91E-06 | 2 | 1.13E+08 | 3018 | 0.3042 | 0.06703 | ebi-a-GCST90001454 | rs74805957 | T | C | 0.0396 | CD62L- HLA DR++ monocyte AC | TRUE | reported | igd | 0.006778 | 20.58221 |
| 8.27E-06 | 3 | 1641354 | 3018 | 0.5986 | 0.134 | ebi-a-GCST90001454 | rs115431394 | C | A | 0.0096 | CD62L- HLA DR++ monocyte AC | TRUE | reported | igd | 0.006569 | 19.94233 |
| 9.92E-06 | 3 | 61569753 | 3018 | 0.1401 | 0.03165 | ebi-a-GCST90001454 | rs1320172 | T | C | 0.2179 | CD62L- HLA DR++ monocyte AC | TRUE | reported | igd | 0.006451 | 19.58127 |
| 8.98E-06 | 4 | 1531162 | 3018 | -0.2927 | 0.06581 | ebi-a-GCST90001454 | rs111224423 | G | C | 0.0409 | CD62L- HLA DR++ monocyte AC | TRUE | reported | igd | 0.006512 | 19.7685 |
| 8.65E-06 | 4 | 1.48E+08 | 3018 | -0.1209 | 0.02713 | ebi-a-GCST90001454 | rs7437802 | T | C | 0.6269 | CD62L- HLA DR++ monocyte AC | TRUE | reported | igd | 0.006537 | 19.84564 |
| 9.53E-06 | 5 | 1.79E+08 | 3018 | 0.1137 | 0.02564 | ebi-a-GCST90001454 | rs3797612 | C | T | 0.4606 | CD62L- HLA DR++ monocyte AC | TRUE | reported | igd | 0.006474 | 19.65156 |
| 6.35E-06 | 6 | 54476928 | 3018 | -0.2094 | 0.0463 | ebi-a-GCST90001454 | rs10948829 | G | A | 0.0911 | CD62L- HLA DR++ monocyte AC | TRUE | reported | igd | 0.006732 | 20.44106 |
| 8.75E-06 | 9 | 1.14E+08 | 3018 | -0.1197 | 0.02688 | ebi-a-GCST90001454 | rs10759491 | T | C | 0.4032 | CD62L- HLA DR++ monocyte AC | TRUE | reported | igd | 0.006528 | 19.81718 |
| 5.90E-06 | 9 | 1.13E+08 | 3018 | -0.1284 | 0.0283 | ebi-a-GCST90001454 | rs10980077 | A | G | 0.3118 | CD62L- HLA DR++ monocyte AC | TRUE | reported | igd | 0.006775 | 20.57166 |
| 8.36E-06 | 10 | 1.24E+08 | 3018 | 0.1276 | 0.02858 | ebi-a-GCST90001454 | rs10887149 | A | G | 0.3101 | CD62L- HLA DR++ monocyte AC | TRUE | reported | igd | 0.006561 | 19.91998 |
| 1.47E-06 | 11 | 1.35E+08 | 3018 | 0.2396 | 0.04966 | ebi-a-GCST90001454 | rs28680389 | A | G | 0.0747 | CD62L- HLA DR++ monocyte AC | TRUE | reported | igd | 0.007654 | 23.26335 |
| 6.94E-07 | 12 | 1.09E+08 | 3018 | 0.1364 | 0.02743 | ebi-a-GCST90001454 | rs938447 | C | T | 0.6473 | CD62L- HLA DR++ monocyte AC | TRUE | reported | igd | 0.008127 | 24.71094 |
| 2.86E-06 | 13 | 76034172 | 3018 | -0.4212 | 0.08981 | ebi-a-GCST90001454 | rs148420500 | A | G | 0.0209 | CD62L- HLA DR++ monocyte AC | TRUE | reported | igd | 0.007235 | 21.98059 |
| 4.53E-06 | 14 | 69321561 | 3018 | 0.1267 | 0.02759 | ebi-a-GCST90001454 | rs4899265 | C | A | 0.3474 | CD62L- HLA DR++ monocyte AC | TRUE | reported | igd | 0.006939 | 21.07473 |
| 2.17E-06 | 14 | 53831558 | 3018 | 0.3934 | 0.08287 | ebi-a-GCST90001454 | rs139554006 | A | G | 0.0255 | CD62L- HLA DR++ monocyte AC | TRUE | reported | igd | 0.007412 | 22.52092 |
| 6.31E-06 | 15 | 88341038 | 3018 | 0.3569 | 0.07891 | ebi-a-GCST90001454 | rs144348353 | T | G | 0.0295 | CD62L- HLA DR++ monocyte AC | TRUE | reported | igd | 0.006732 | 20.44283 |
| 7.06E-06 | 16 | 50273295 | 3018 | -0.1416 | 0.03148 | ebi-a-GCST90001454 | rs7190107 | A | T | 0.2169 | CD62L- HLA DR++ monocyte AC | TRUE | reported | igd | 0.006659 | 20.21944 |
| 8.89E-06 | 18 | 3595011 | 3018 | -0.1231 | 0.02767 | ebi-a-GCST90001454 | rs2240899 | C | G | 0.6678 | CD62L- HLA DR++ monocyte AC | TRUE | reported | igd | 0.006515 | 19.77925 |
| 5.05E-07 | 22 | 25622885 | 3018 | 0.1995 | 0.03962 | ebi-a-GCST90001454 | rs117545845 | T | C | 0.1209 | CD62L- HLA DR++ monocyte AC | TRUE | reported | igd | 0.008331 | 25.3378 |
| 7.10E-06 | 1 | 1.65E+08 | 1858 | 0.2421 | 0.05377 | ebi-a-GCST90001519 | rs10494422 | C | A | 0.1074 | CD33br HLA DR+ CD14- %CD33br HLA DR+ | TRUE | reported | igd | 0.010793 | 20.25078 |
| 9.41E-06 | 1 | 2.28E+08 | 1858 | -0.1496 | 0.03367 | ebi-a-GCST90001519 | rs10799466 | A | G | 0.5067 | CD33br HLA DR+ CD14- %CD33br HLA DR+ | TRUE | reported | igd | 0.010513 | 19.7201 |
| 5.40E-08 | 1 | 85552939 | 1858 | -0.262 | 0.04799 | ebi-a-GCST90001519 | rs12033128 | C | T | 0.1389 | CD33br HLA DR+ CD14- %CD33br HLA DR+ | TRUE | reported | igd | 0.015789 | 29.77374 |
| 2.32E-06 | 2 | 6895049 | 1858 | -0.196 | 0.04137 | ebi-a-GCST90001519 | rs16865573 | C | T | 0.2199 | CD33br HLA DR+ CD14- %CD33br HLA DR+ | TRUE | reported | igd | 0.011937 | 22.42195 |
| 3.58E-06 | 2 | 1.42E+08 | 1858 | 0.4192 | 0.09019 | ebi-a-GCST90001519 | rs116534482 | C | T | 0.0342 | CD33br HLA DR+ CD14- %CD33br HLA DR+ | TRUE | reported | igd | 0.011494 | 21.58033 |
| 9.57E-07 | 4 | 14437080 | 1858 | 0.3392 | 0.06898 | ebi-a-GCST90001519 | rs218773 | G | A | 0.937 | CD33br HLA DR+ CD14- %CD33br HLA DR+ | TRUE | reported | igd | 0.012847 | 24.15447 |
| 5.20E-06 | 4 | 36025181 | 1858 | -0.2317 | 0.05069 | ebi-a-GCST90001519 | rs115205981 | A | G | 0.1243 | CD33br HLA DR+ CD14- %CD33br HLA DR+ | TRUE | reported | igd | 0.01112 | 20.87083 |
| 2.83E-06 | 4 | 1.91E+08 | 1858 | -0.4891 | 0.1041 | ebi-a-GCST90001519 | rs6844114 | A | G | 0.0269 | CD33br HLA DR+ CD14- %CD33br HLA DR+ | TRUE | reported | igd | 0.011741 | 22.05089 |
| 3.14E-06 | 4 | 1.65E+08 | 1858 | 1.103 | 0.236 | ebi-a-GCST90001519 | rs62350505 | G | C | 0.0054 | CD33br HLA DR+ CD14- %CD33br HLA DR+ | TRUE | reported | igd | 0.01162 | 21.82023 |
| 5.75E-06 | 6 | 90899561 | 1858 | 0.1697 | 0.0373 | ebi-a-GCST90001519 | rs2501720 | A | G | 0.267 | CD33br HLA DR+ CD14- %CD33br HLA DR+ | TRUE | reported | igd | 0.011018 | 20.67656 |
| 9.16E-06 | 7 | 67501241 | 1858 | 0.1825 | 0.04102 | ebi-a-GCST90001519 | rs11982860 | A | G | 0.2096 | CD33br HLA DR+ CD14- %CD33br HLA DR+ | TRUE | reported | igd | 0.010541 | 19.77273 |
| 5.71E-06 | 7 | 1.4E+08 | 1858 | -1.297 | 0.2851 | ebi-a-GCST90001519 | rs142412842 | C | A | 0.004 | CD33br HLA DR+ CD14- %CD33br HLA DR+ | TRUE | reported | igd | 0.011016 | 20.67368 |
| 7.83E-06 | 9 | 83651275 | 1858 | 2.17 | 0.4841 | ebi-a-GCST90001519 | rs111391334 | G | A | 0.0013 | CD33br HLA DR+ CD14- %CD33br HLA DR+ | TRUE | reported | igd | 0.010699 | 20.07158 |
| 4.43E-06 | 11 | 35244058 | 1858 | -0.1819 | 0.03952 | ebi-a-GCST90001519 | rs7116432 | G | A | 0.2333 | CD33br HLA DR+ CD14- %CD33br HLA DR+ | TRUE | reported | igd | 0.011274 | 21.16234 |
| 6.02E-07 | 17 | 12239004 | 1858 | 0.2193 | 0.0438 | ebi-a-GCST90001519 | rs2529690 | A | G | 0.1747 | CD33br HLA DR+ CD14- %CD33br HLA DR+ | TRUE | reported | igd | 0.013313 | 25.04156 |
| 3.02E-06 | 18 | 70222212 | 1858 | -1.707 | 0.3645 | ebi-a-GCST90001519 | rs113548770 | G | A | 0.0019 | CD33br HLA DR+ CD14- %CD33br HLA DR+ | TRUE | reported | igd | 0.011666 | 21.90808 |
| 2.63E-15 | 19 | 51731176 | 1858 | -0.3128 | 0.03922 | ebi-a-GCST90001519 | rs7245846 | A | G | 0.2236 | CD33br HLA DR+ CD14- %CD33br HLA DR+ | TRUE | reported | igd | 0.033102 | 63.54049 |
| 7.57E-06 | 19 | 51897760 | 1858 | -0.2749 | 0.06122 | ebi-a-GCST90001519 | rs111695695 | A | C | 0.0823 | CD33br HLA DR+ CD14- %CD33br HLA DR+ | TRUE | reported | igd | 0.010736 | 20.14165 |
| 4.02E-06 | 20 | 61646108 | 1858 | -0.2378 | 0.05142 | ebi-a-GCST90001519 | rs62197298 | T | C | 0.1173 | CD33br HLA DR+ CD14- %CD33br HLA DR+ | TRUE | reported | igd | 0.01138 | 21.36445 |
| 1.65E-06 | 1 | 1.57E+08 | 3395 | 0.1224 | 0.0255 | ebi-a-GCST90001537 | rs12043060 | T | C | 0.4633 | CM CD4+ AC | TRUE | reported | igd | 0.006741 | 23.02643 |
| 3.83E-07 | 1 | 1.98E+08 | 3395 | -1.329 | 0.2612 | ebi-a-GCST90001537 | rs541367304 | A | G | 0.0032 | CM CD4+ AC | TRUE | reported | igd | 0.007568 | 25.87305 |
| 5.73E-06 | 2 | 38920333 | 3395 | 0.1343 | 0.02955 | ebi-a-GCST90001537 | rs10206629 | C | T | 0.2443 | CM CD4+ AC | TRUE | reported | igd | 0.006047 | 20.6434 |
| 9.06E-06 | 3 | 75433850 | 3395 | -0.1204 | 0.02708 | ebi-a-GCST90001537 | rs2875222 | A | G | 0.5577 | CM CD4+ AC | TRUE | reported | igd | 0.005789 | 19.75603 |
| 3.63E-06 | 4 | 64094440 | 3395 | 1.777 | 0.383 | ebi-a-GCST90001537 | rs79668094 | A | G | 0.001 | CM CD4+ AC | TRUE | reported | igd | 0.006301 | 21.51401 |
| 7.74E-06 | 4 | 94012466 | 3395 | 0.3714 | 0.08292 | ebi-a-GCST90001537 | rs11725186 | A | T | 0.0233 | CM CD4+ AC | TRUE | reported | igd | 0.005874 | 20.04977 |
| 1.07E-07 | 5 | 1.78E+08 | 3395 | -0.6098 | 0.1145 | ebi-a-GCST90001537 | rs76664097 | A | G | 0.0115 | CM CD4+ AC | TRUE | reported | igd | 0.008285 | 28.34706 |
| 1.83E-07 | 6 | 32591845 | 3395 | 0.1821 | 0.03484 | ebi-a-GCST90001537 | rs9271622 | A | G | 0.7477 | CM CD4+ AC | TRUE | reported | igd | 0.007983 | 27.30283 |
| 2.98E-06 | 6 | 32586922 | 3395 | -0.1483 | 0.03169 | ebi-a-GCST90001537 | rs13207893 | C | T | 0.2954 | CM CD4+ AC | TRUE | reported | igd | 0.006409 | 21.88678 |
| 4.42E-06 | 6 | 1.49E+08 | 3395 | -0.2038 | 0.04433 | ebi-a-GCST90001537 | rs34327811 | C | T | 0.0887 | CM CD4+ AC | TRUE | reported | igd | 0.006187 | 21.12307 |
| 1.87E-06 | 6 | 7780498 | 3395 | 0.2519 | 0.05276 | ebi-a-GCST90001537 | rs75024452 | T | A | 0.0617 | CM CD4+ AC | TRUE | reported | igd | 0.00667 | 22.78195 |
| 5.77E-06 | 6 | 1.02E+08 | 3395 | 0.2327 | 0.05124 | ebi-a-GCST90001537 | rs7760353 | A | T | 0.0663 | CM CD4+ AC | TRUE | reported | igd | 0.006038 | 20.61193 |
| 1.87E-07 | 7 | 18003774 | 3395 | 0.4171 | 0.07987 | ebi-a-GCST90001537 | rs17717645 | C | T | 0.0267 | CM CD4+ AC | TRUE | reported | igd | 0.007969 | 27.25568 |
| 8.64E-06 | 8 | 17277790 | 3395 | 0.2507 | 0.05627 | ebi-a-GCST90001537 | rs11990065 | G | A | 0.0539 | CM CD4+ AC | TRUE | reported | igd | 0.005813 | 19.83805 |
| 8.59E-06 | 9 | 27446846 | 3395 | -0.2997 | 0.06724 | ebi-a-GCST90001537 | rs118048878 | A | G | 0.0368 | CM CD4+ AC | TRUE | reported | igd | 0.005818 | 19.85465 |
| 2.87E-06 | 12 | 65373483 | 3395 | 0.6116 | 0.1305 | ebi-a-GCST90001537 | rs183058497 | C | G | 0.0091 | CM CD4+ AC | TRUE | reported | igd | 0.006428 | 21.95119 |
| 5.40E-06 | 12 | 20885314 | 3395 | -0.667 | 0.1464 | ebi-a-GCST90001537 | rs1913821 | C | T | 0.9922 | CM CD4+ AC | TRUE | reported | igd | 0.006077 | 20.74501 |
| 2.98E-09 | 12 | 1.12E+08 | 3395 | -0.1609 | 0.02705 | ebi-a-GCST90001537 | rs3184504 | C | T | 0.5292 | CM CD4+ AC | TRUE | reported | igd | 0.010314 | 35.36076 |
| 4.98E-06 | 12 | 3830558 | 3395 | 0.1592 | 0.03482 | ebi-a-GCST90001537 | rs11062774 | G | A | 0.1558 | CM CD4+ AC | TRUE | reported | igd | 0.00612 | 20.89165 |
| 7.65E-08 | 12 | 16811638 | 3395 | 0.4832 | 0.08969 | ebi-a-GCST90001537 | rs151245760 | C | T | 0.0206 | CM CD4+ AC | TRUE | reported | igd | 0.008477 | 29.00747 |
| 3.84E-06 | 12 | 26948822 | 3395 | -0.4084 | 0.08826 | ebi-a-GCST90001537 | rs55873755 | T | C | 0.0209 | CM CD4+ AC | TRUE | reported | igd | 0.006267 | 21.39872 |
| 8.03E-06 | 13 | 1.01E+08 | 3395 | -0.1525 | 0.0341 | ebi-a-GCST90001537 | rs1980708 | C | T | 0.8293 | CM CD4+ AC | TRUE | reported | igd | 0.005857 | 19.98826 |
| 6.84E-06 | 15 | 97948444 | 3395 | 0.2554 | 0.05668 | ebi-a-GCST90001537 | rs78720100 | T | C | 0.0495 | CM CD4+ AC | TRUE | reported | igd | 0.005945 | 20.29206 |
| 4.48E-06 | 15 | 26030485 | 3395 | -0.1159 | 0.02523 | ebi-a-GCST90001537 | rs7162344 | A | G | 0.4315 | CM CD4+ AC | TRUE | reported | igd | 0.006177 | 21.08999 |
| 9.04E-06 | 17 | 7887429 | 3395 | -0.2959 | 0.06656 | ebi-a-GCST90001537 | rs117066544 | A | G | 0.0387 | CM CD4+ AC | TRUE | reported | igd | 0.005788 | 19.75183 |
| 4.09E-06 | 17 | 72909337 | 3395 | 0.1872 | 0.04058 | ebi-a-GCST90001537 | rs144079997 | T | C | 0.1081 | CM CD4+ AC | TRUE | reported | igd | 0.006229 | 21.26825 |
| 1.18E-06 | 20 | 56223607 | 3395 | 0.3231 | 0.06637 | ebi-a-GCST90001537 | rs1043118 | A | G | 0.0376 | CM CD4+ AC | TRUE | reported | igd | 0.006932 | 23.68505 |
| 8.44E-06 | 20 | 10891892 | 3395 | -0.2251 | 0.05047 | ebi-a-GCST90001537 | rs61148192 | G | A | 0.0645 | CM CD4+ AC | TRUE | reported | igd | 0.005825 | 19.88055 |
| 6.42E-06 | 21 | 33131559 | 3395 | 0.4463 | 0.09875 | ebi-a-GCST90001537 | rs17584809 | T | C | 0.0169 | CM CD4+ AC | TRUE | reported | igd | 0.00598 | 20.41379 |
| 9.07E-06 | 1 | 2.14E+08 | 3652 | -0.1735 | 0.03904 | ebi-a-GCST90001589 | rs11120194 | C | T | 0.1113 | CD4/CD8br | TRUE | reported | igd | 0.005379 | 19.73974 |
| 7.83E-06 | 1 | 2.42E+08 | 3652 | 0.1564 | 0.03494 | ebi-a-GCST90001589 | rs12139740 | T | C | 0.1465 | CD4/CD8br | TRUE | reported | igd | 0.005457 | 20.0258 |
| 7.34E-06 | 1 | 19649970 | 3652 | -0.2075 | 0.04621 | ebi-a-GCST90001589 | rs141317121 | A | G | 0.0772 | CD4/CD8br | TRUE | reported | igd | 0.005491 | 20.15238 |
| 3.48E-06 | 2 | 1.14E+08 | 3652 | -0.1442 | 0.03102 | ebi-a-GCST90001589 | rs13418326 | G | C | 0.2063 | CD4/CD8br | TRUE | reported | igd | 0.005882 | 21.59778 |
| 3.56E-06 | 4 | 1685211 | 3652 | -0.1395 | 0.03005 | ebi-a-GCST90001589 | rs798726 | C | T | 0.7809 | CD4/CD8br | TRUE | reported | igd | 0.005866 | 21.5388 |
| 2.04E-06 | 6 | 1.17E+08 | 3652 | 0.2925 | 0.06149 | ebi-a-GCST90001589 | rs955275 | G | A | 0.0435 | CD4/CD8br | TRUE | reported | igd | 0.006158 | 22.61543 |
| 2.39E-08 | 6 | 31388449 | 3652 | 0.1905 | 0.03406 | ebi-a-GCST90001589 | rs73402222 | C | T | 0.3456 | CD4/CD8br | TRUE | reported | igd | 0.008493 | 31.26531 |
| 7.10E-06 | 6 | 31414327 | 3652 | -0.1347 | 0.02995 | ebi-a-GCST90001589 | rs2516466 | G | T | 0.3758 | CD4/CD8br | TRUE | reported | igd | 0.005508 | 20.21639 |
| 4.36E-06 | 7 | 1.39E+08 | 3652 | 0.8545 | 0.1858 | ebi-a-GCST90001589 | rs74493069 | A | G | 0.0041 | CD4/CD8br | TRUE | reported | igd | 0.005758 | 21.1395 |
| 7.64E-06 | 7 | 9337459 | 3652 | 0.4252 | 0.09488 | ebi-a-GCST90001589 | rs1529247 | A | T | 0.983 | CD4/CD8br | TRUE | reported | igd | 0.005469 | 20.0724 |
| 7.98E-06 | 9 | 99054924 | 3652 | -0.117 | 0.02616 | ebi-a-GCST90001589 | rs10990269 | T | C | 0.3527 | CD4/CD8br | TRUE | reported | igd | 0.005447 | 19.9921 |
| 1.64E-06 | 10 | 90271135 | 3652 | 0.1243 | 0.02588 | ebi-a-GCST90001589 | rs2576166 | G | C | 0.553 | CD4/CD8br | TRUE | reported | igd | 0.006277 | 23.05557 |
| 7.37E-06 | 10 | 74476968 | 3652 | 0.4504 | 0.1003 | ebi-a-GCST90001589 | rs117942997 | G | C | 0.0189 | CD4/CD8br | TRUE | reported | igd | 0.005491 | 20.1538 |
| 3.10E-06 | 17 | 74440890 | 3652 | 0.3049 | 0.06527 | ebi-a-GCST90001589 | rs495055 | G | A | 0.0357 | CD4/CD8br | TRUE | reported | igd | 0.00594 | 21.8097 |
| 7.37E-06 | 17 | 33774635 | 3652 | 0.1148 | 0.02558 | ebi-a-GCST90001589 | rs11080350 | T | C | 0.4018 | CD4/CD8br | TRUE | reported | igd | 0.005485 | 20.13005 |
| 3.26E-06 | 19 | 1671549 | 3652 | -0.121 | 0.02596 | ebi-a-GCST90001589 | rs62130076 | A | G | 0.3472 | CD4/CD8br | TRUE | reported | igd | 0.005914 | 21.71318 |
| 4.24E-06 | 1 | 1.03E+08 | 3668 | 0.9785 | 0.2124 | ebi-a-GCST90001593 | rs76595423 | G | T | 0.0037 | CD8br %T cell | TRUE | reported | igd | 0.005753 | 21.21171 |
| 5.63E-06 | 1 | 19649970 | 3668 | 0.2079 | 0.04572 | ebi-a-GCST90001593 | rs141317121 | A | G | 0.077 | CD8br %T cell | TRUE | reported | igd | 0.005606 | 20.66615 |
| 3.59E-06 | 1 | 2.42E+08 | 3668 | -0.1603 | 0.03454 | ebi-a-GCST90001593 | rs12139740 | T | C | 0.1465 | CD8br %T cell | TRUE | reported | igd | 0.005838 | 21.5271 |
| 8.24E-07 | 2 | 1.14E+08 | 3668 | 0.1508 | 0.03053 | ebi-a-GCST90001593 | rs13418326 | G | C | 0.2058 | CD8br %T cell | TRUE | reported | igd | 0.006608 | 24.38441 |
| 7.71E-06 | 4 | 41910384 | 3668 | -0.2001 | 0.04467 | ebi-a-GCST90001593 | rs7670980 | T | G | 0.0841 | CD8br %T cell | TRUE | reported | igd | 0.005441 | 20.05513 |
| 5.76E-06 | 4 | 1685211 | 3668 | 0.135 | 0.02972 | ebi-a-GCST90001593 | rs798726 | C | T | 0.7814 | CD8br %T cell | TRUE | reported | igd | 0.005594 | 20.62211 |
| 6.62E-06 | 5 | 9372480 | 3668 | -0.1132 | 0.02509 | ebi-a-GCST90001593 | rs40653 | T | C | 0.613 | CD8br %T cell | TRUE | reported | igd | 0.005519 | 20.34486 |
| 5.74E-06 | 5 | 71408867 | 3668 | 0.135 | 0.02972 | ebi-a-GCST90001593 | rs2337390 | G | A | 0.7927 | CD8br %T cell | TRUE | reported | igd | 0.005594 | 20.62211 |
| 2.76E-09 | 6 | 31388449 | 3668 | -0.1934 | 0.03245 | ebi-a-GCST90001593 | rs73402222 | C | T | 0.3458 | CD8br %T cell | TRUE | reported | igd | 0.009591 | 35.5015 |
| 1.26E-06 | 6 | 31414327 | 3668 | 0.1401 | 0.02887 | ebi-a-GCST90001593 | rs2516466 | G | T | 0.3762 | CD8br %T cell | TRUE | reported | igd | 0.006379 | 23.53671 |
| 1.53E-06 | 6 | 1.17E+08 | 3668 | -0.2919 | 0.06061 | ebi-a-GCST90001593 | rs955275 | G | A | 0.0433 | CD8br %T cell | TRUE | reported | igd | 0.006284 | 23.18157 |
| 9.44E-06 | 7 | 1.39E+08 | 3668 | -0.8197 | 0.1848 | ebi-a-GCST90001593 | rs74493069 | A | G | 0.0041 | CD8br %T cell | TRUE | reported | igd | 0.005335 | 19.66387 |
| 5.47E-06 | 7 | 67315960 | 3668 | 0.2698 | 0.05927 | ebi-a-GCST90001593 | rs62458311 | A | G | 0.0447 | CD8br %T cell | TRUE | reported | igd | 0.005617 | 20.70986 |
| 8.91E-06 | 8 | 61782205 | 3668 | -0.3983 | 0.08953 | ebi-a-GCST90001593 | rs7826289 | T | C | 0.0188 | CD8br %T cell | TRUE | reported | igd | 0.005367 | 19.78092 |
| 1.91E-06 | 9 | 99054924 | 3668 | 0.1232 | 0.02583 | ebi-a-GCST90001593 | rs10990269 | T | C | 0.3525 | CD8br %T cell | TRUE | reported | igd | 0.006164 | 22.73713 |
| 6.65E-06 | 10 | 76644830 | 3668 | -0.2386 | 0.0529 | ebi-a-GCST90001593 | rs116943003 | A | T | 0.0624 | CD8br %T cell | TRUE | reported | igd | 0.005516 | 20.33259 |
| 9.19E-06 | 10 | 74476968 | 3668 | -0.4398 | 0.09901 | ebi-a-GCST90001593 | rs117942997 | G | C | 0.0189 | CD8br %T cell | TRUE | reported | igd | 0.00535 | 19.72039 |
| 1.89E-06 | 10 | 90271135 | 3668 | -0.121 | 0.02535 | ebi-a-GCST90001593 | rs2576166 | G | C | 0.5532 | CD8br %T cell | TRUE | reported | igd | 0.006173 | 22.77078 |
| 9.50E-06 | 11 | 1.3E+08 | 3668 | -0.372 | 0.08388 | ebi-a-GCST90001593 | rs74381175 | T | G | 0.0211 | CD8br %T cell | TRUE | reported | igd | 0.005334 | 19.65768 |
| 3.33E-06 | 11 | 74879130 | 3668 | 0.1876 | 0.04029 | ebi-a-GCST90001593 | rs1676877 | G | A | 0.8993 | CD8br %T cell | TRUE | reported | igd | 0.005876 | 21.66877 |
| 8.02E-06 | 16 | 13172942 | 3668 | -0.1374 | 0.03073 | ebi-a-GCST90001593 | rs12449027 | A | G | 0.1884 | CD8br %T cell | TRUE | reported | igd | 0.005421 | 19.98074 |
| 4.24E-06 | 17 | 74440890 | 3668 | -0.2968 | 0.06444 | ebi-a-GCST90001593 | rs495055 | G | A | 0.0357 | CD8br %T cell | TRUE | reported | igd | 0.00575 | 21.20215 |
| 1.61E-06 | 19 | 1671549 | 3668 | 0.1233 | 0.02565 | ebi-a-GCST90001593 | rs62130076 | A | G | 0.3472 | CD8br %T cell | TRUE | reported | igd | 0.00626 | 23.09482 |
| 7.81E-06 | 19 | 16748884 | 3668 | -0.2993 | 0.06686 | ebi-a-GCST90001593 | rs73516994 | T | C | 0.0348 | CD8br %T cell | TRUE | reported | igd | 0.005434 | 20.02829 |
| 2.25E-06 | 2 | 13251154 | 3596 | 0.548 | 0.1157 | ebi-a-GCST90001619 | rs79178843 | A | G | 0.0097 | HLA DR+ T cell%T cell | TRUE | reported | igd | 0.0062 | 22.42089 |
| 9.51E-06 | 2 | 1.59E+08 | 3596 | -0.3105 | 0.07001 | ebi-a-GCST90001619 | rs116797692 | A | G | 0.0292 | HLA DR+ T cell%T cell | TRUE | reported | igd | 0.00544 | 19.659 |
| 7.65E-06 | 3 | 74944494 | 3596 | 2.031 | 0.4533 | ebi-a-GCST90001619 | rs145794521 | A | G | 6.00E-04 | HLA DR+ T cell%T cell | TRUE | reported | igd | 0.005552 | 20.0635 |
| 9.80E-07 | 4 | 27183687 | 3596 | 0.3143 | 0.06409 | ebi-a-GCST90001619 | rs148323639 | A | G | 0.0317 | HLA DR+ T cell%T cell | TRUE | reported | igd | 0.006643 | 24.03624 |
| 6.53E-06 | 6 | 50683009 | 3596 | -0.1411 | 0.03125 | ebi-a-GCST90001619 | rs78648104 | C | T | 0.1714 | HLA DR+ T cell%T cell | TRUE | reported | igd | 0.005637 | 20.37569 |
| 9.31E-09 | 6 | 32582612 | 3596 | 0.1693 | 0.0294 | ebi-a-GCST90001619 | rs3104414 | A | G | 0.3813 | HLA DR+ T cell%T cell | TRUE | reported | igd | 0.009137 | 33.14192 |
| 3.08E-06 | 7 | 46432552 | 3596 | -1.414 | 0.3025 | ebi-a-GCST90001619 | rs10244964 | T | G | 0.0013 | HLA DR+ T cell%T cell | TRUE | reported | igd | 0.006039 | 21.83768 |
| 8.21E-06 | 7 | 1.15E+08 | 3596 | -0.3744 | 0.08384 | ebi-a-GCST90001619 | rs76997602 | G | A | 0.0178 | HLA DR+ T cell%T cell | TRUE | reported | igd | 0.005515 | 19.93093 |
| 9.53E-06 | 8 | 11817727 | 3596 | -0.7017 | 0.1583 | ebi-a-GCST90001619 | rs138678763 | C | T | 0.005 | HLA DR+ T cell%T cell | TRUE | reported | igd | 0.005434 | 19.6381 |
| 7.82E-06 | 8 | 1.05E+08 | 3596 | -0.5382 | 0.1202 | ebi-a-GCST90001619 | rs117941717 | G | A | 0.009 | HLA DR+ T cell%T cell | TRUE | reported | igd | 0.005544 | 20.03719 |
| 8.94E-06 | 9 | 1.02E+08 | 3596 | 0.1033 | 0.02322 | ebi-a-GCST90001619 | rs7047722 | A | G | 0.6279 | HLA DR+ T cell%T cell | TRUE | reported | igd | 0.005474 | 19.78038 |
| 5.19E-06 | 9 | 1.01E+08 | 3596 | 0.2231 | 0.04888 | ebi-a-GCST90001619 | rs144674286 | A | G | 0.0628 | HLA DR+ T cell%T cell | TRUE | reported | igd | 0.00576 | 20.82069 |
| 1.46E-09 | 9 | 1.18E+08 | 3596 | -0.1471 | 0.02426 | ebi-a-GCST90001619 | rs7032773 | T | C | 0.4182 | HLA DR+ T cell%T cell | TRUE | reported | igd | 0.010121 | 36.74533 |
| 5.39E-06 | 9 | 22725624 | 3596 | 0.1347 | 0.02956 | ebi-a-GCST90001619 | rs687184 | C | G | 0.8184 | HLA DR+ T cell%T cell | TRUE | reported | igd | 0.005741 | 20.75318 |
| 2.25E-06 | 10 | 76784567 | 3596 | -0.9683 | 0.2044 | ebi-a-GCST90001619 | rs117014352 | T | C | 0.0028 | HLA DR+ T cell%T cell | TRUE | reported | igd | 0.006202 | 22.42934 |
| 4.95E-06 | 10 | 1.22E+08 | 3596 | 0.2157 | 0.04716 | ebi-a-GCST90001619 | rs10788105 | A | G | 0.0601 | HLA DR+ T cell%T cell | TRUE | reported | igd | 0.005784 | 20.90793 |
| 2.43E-06 | 10 | 3066899 | 3596 | -0.1849 | 0.03916 | ebi-a-GCST90001619 | rs7088079 | A | G | 0.0891 | HLA DR+ T cell%T cell | TRUE | reported | igd | 0.006161 | 22.28162 |
| 7.74E-06 | 11 | 86071947 | 3596 | 0.1021 | 0.0228 | ebi-a-GCST90001619 | rs7127620 | G | A | 0.46 | HLA DR+ T cell%T cell | TRUE | reported | igd | 0.005546 | 20.04196 |
| 8.19E-07 | 11 | 1.08E+08 | 3596 | -0.1125 | 0.02277 | ebi-a-GCST90001619 | rs542160 | T | C | 0.5089 | HLA DR+ T cell%T cell | TRUE | reported | igd | 0.006743 | 24.39705 |
| 9.23E-06 | 11 | 1.07E+08 | 3596 | 0.1937 | 0.04361 | ebi-a-GCST90001619 | rs79132531 | G | A | 0.0749 | HLA DR+ T cell%T cell | TRUE | reported | igd | 0.005456 | 19.71721 |
| 9.23E-06 | 12 | 1.27E+08 | 3596 | 0.1028 | 0.02315 | ebi-a-GCST90001619 | rs7294683 | G | A | 0.3899 | HLA DR+ T cell%T cell | TRUE | reported | igd | 0.005454 | 19.708 |
| 3.70E-06 | 12 | 12457054 | 3596 | 0.2888 | 0.06232 | ebi-a-GCST90001619 | rs11054779 | T | G | 0.0331 | HLA DR+ T cell%T cell | TRUE | reported | igd | 0.005937 | 21.46337 |
| 7.56E-06 | 13 | 51299267 | 3596 | 0.4774 | 0.1065 | ebi-a-GCST90001619 | rs9526689 | A | G | 0.0106 | HLA DR+ T cell%T cell | TRUE | reported | igd | 0.005557 | 20.08279 |
| 2.64E-06 | 13 | 1.11E+08 | 3596 | -0.3833 | 0.08148 | ebi-a-GCST90001619 | rs506151 | T | C | 0.02 | HLA DR+ T cell%T cell | TRUE | reported | igd | 0.006116 | 22.1174 |
| 3.08E-70 | 16 | 10970902 | 3596 | -0.4611 | 0.02546 | ebi-a-GCST90001619 | rs3087456 | A | G | 0.7661 | HLA DR+ T cell%T cell | TRUE | reported | igd | 0.083588 | 327.8173 |
| 1.90E-06 | 16 | 11357860 | 3596 | -0.245 | 0.05135 | ebi-a-GCST90001619 | rs71381201 | G | T | 0.0517 | HLA DR+ T cell%T cell | TRUE | reported | igd | 0.006291 | 22.75148 |
| 9.71E-09 | 16 | 10824872 | 3596 | -0.1328 | 0.02309 | ebi-a-GCST90001619 | rs1084560 | G | A | 0.5726 | HLA DR+ T cell%T cell | TRUE | reported | igd | 0.009115 | 33.06029 |
| 9.89E-08 | 16 | 10993712 | 3596 | -0.2435 | 0.0456 | ebi-a-GCST90001619 | rs55724950 | A | G | 0.0654 | HLA DR+ T cell%T cell | TRUE | reported | igd | 0.007867 | 28.4988 |
| 5.73E-06 | 17 | 11457702 | 3596 | -0.1065 | 0.02345 | ebi-a-GCST90001619 | rs12601032 | A | G | 0.3598 | HLA DR+ T cell%T cell | TRUE | reported | igd | 0.005703 | 20.61446 |
| 8.05E-06 | 17 | 50420836 | 3596 | -0.3778 | 0.08451 | ebi-a-GCST90001619 | rs62068717 | T | C | 0.0204 | HLA DR+ T cell%T cell | TRUE | reported | igd | 0.005527 | 19.97405 |
| 1.39E-06 | 18 | 55985546 | 3596 | 1.483 | 0.3068 | ebi-a-GCST90001619 | rs138213638 | C | T | 0.0014 | HLA DR+ T cell%T cell | TRUE | reported | igd | 0.006456 | 23.35232 |
| 7.90E-07 | 19 | 1671156 | 3596 | -0.2381 | 0.04814 | ebi-a-GCST90001619 | rs140410174 | T | C | 0.0598 | HLA DR+ T cell%T cell | TRUE | reported | igd | 0.006757 | 24.44922 |
| 3.95E-07 | 19 | 38996871 | 3596 | 1.729 | 0.3404 | ebi-a-GCST90001619 | rs73030989 | T | C | 0.0013 | HLA DR+ T cell%T cell | TRUE | reported | igd | 0.007123 | 25.78513 |
| 1.53E-06 | 1 | 1220504 | 3579 | -0.3548 | 0.07367 | ebi-a-GCST90001624 | rs2064600 | A | G | 0.0295 | HLA DR+ CD4+ AC | TRUE | reported | igd | 0.006439 | 23.18158 |
| 6.76E-06 | 2 | 1.21E+08 | 3579 | -0.4445 | 0.09861 | ebi-a-GCST90001624 | rs62166973 | A | G | 0.0148 | HLA DR+ CD4+ AC | TRUE | reported | igd | 0.005645 | 20.30761 |
| 2.78E-06 | 3 | 1.8E+08 | 3579 | 0.1335 | 0.02844 | ebi-a-GCST90001624 | rs6767083 | A | C | 0.2311 | HLA DR+ CD4+ AC | TRUE | reported | igd | 0.006119 | 22.02219 |
| 3.69E-06 | 3 | 75122371 | 3579 | -0.1129 | 0.02435 | ebi-a-GCST90001624 | rs55849757 | T | C | 0.5545 | HLA DR+ CD4+ AC | TRUE | reported | igd | 0.005971 | 21.48559 |
| 9.64E-06 | 3 | 5810961 | 3579 | -0.9842 | 0.2221 | ebi-a-GCST90001624 | rs17824379 | G | A | 0.0027 | HLA DR+ CD4+ AC | TRUE | reported | igd | 0.005457 | 19.62578 |
| 5.62E-06 | 5 | 1.55E+08 | 3579 | 0.3491 | 0.07676 | ebi-a-GCST90001624 | rs6891593 | A | G | 0.0239 | HLA DR+ CD4+ AC | TRUE | reported | igd | 0.005746 | 20.67221 |
| 2.23E-06 | 6 | 32674059 | 3579 | 0.3737 | 0.07884 | ebi-a-GCST90001624 | rs3134968 | G | A | 0.9761 | HLA DR+ CD4+ AC | TRUE | reported | igd | 0.006238 | 22.45485 |
| 1.93E-06 | 6 | 31266015 | 3579 | -0.2281 | 0.04783 | ebi-a-GCST90001624 | rs17198888 | G | A | 0.0802 | HLA DR+ CD4+ AC | TRUE | reported | igd | 0.006314 | 22.7304 |
| 5.67E-06 | 6 | 1.14E+08 | 3579 | -0.1396 | 0.03071 | ebi-a-GCST90001624 | rs62417423 | G | A | 0.1949 | HLA DR+ CD4+ AC | TRUE | reported | igd | 0.00574 | 20.6523 |
| 9.98E-06 | 7 | 70660195 | 3579 | -0.1766 | 0.03991 | ebi-a-GCST90001624 | rs79769908 | G | T | 0.1023 | HLA DR+ CD4+ AC | TRUE | reported | igd | 0.005441 | 19.5693 |
| 7.03E-06 | 8 | 1690475 | 3579 | -0.1662 | 0.03693 | ebi-a-GCST90001624 | rs2977197 | C | G | 0.8806 | HLA DR+ CD4+ AC | TRUE | reported | igd | 0.005627 | 20.24234 |
| 4.75E-09 | 9 | 1.18E+08 | 3579 | -0.1511 | 0.02575 | ebi-a-GCST90001624 | rs1322067 | G | A | 0.4173 | HLA DR+ CD4+ AC | TRUE | reported | igd | 0.009529 | 34.41373 |
| 6.14E-06 | 10 | 1.18E+08 | 3579 | -0.1963 | 0.04335 | ebi-a-GCST90001624 | rs10787670 | C | T | 0.9173 | HLA DR+ CD4+ AC | TRUE | reported | igd | 0.005697 | 20.49367 |
| 3.23E-06 | 10 | 11713685 | 3579 | 0.1603 | 0.03437 | ebi-a-GCST90001624 | rs78229126 | A | C | 0.1357 | HLA DR+ CD4+ AC | TRUE | reported | igd | 0.006041 | 21.74028 |
| 6.44E-06 | 10 | 3327039 | 3579 | 0.1894 | 0.04192 | ebi-a-GCST90001624 | rs11251832 | A | G | 0.0884 | HLA DR+ CD4+ AC | TRUE | reported | igd | 0.005671 | 20.40209 |
| 6.03E-06 | 12 | 14382617 | 3579 | 0.1584 | 0.03494 | ebi-a-GCST90001624 | rs10845930 | C | T | 0.8639 | HLA DR+ CD4+ AC | TRUE | reported | igd | 0.00571 | 20.54101 |
| 7.63E-07 | 14 | 20261108 | 3579 | -0.2544 | 0.05136 | ebi-a-GCST90001624 | rs10148838 | C | G | 0.227 | HLA DR+ CD4+ AC | TRUE | reported | igd | 0.006809 | 24.52118 |
| 3.33E-06 | 14 | 80335426 | 3579 | 0.1473 | 0.03164 | ebi-a-GCST90001624 | rs7150202 | T | A | 0.1707 | HLA DR+ CD4+ AC | TRUE | reported | igd | 0.006019 | 21.66156 |
| 9.29E-06 | 15 | 45945654 | 3579 | -0.783 | 0.1764 | ebi-a-GCST90001624 | rs117110835 | A | G | 0.0047 | HLA DR+ CD4+ AC | TRUE | reported | igd | 0.005475 | 19.69172 |
| 5.86E-06 | 15 | 80129450 | 3579 | -2.588 | 0.5703 | ebi-a-GCST90001624 | rs142283210 | G | T | 4.00E-04 | HLA DR+ CD4+ AC | TRUE | reported | igd | 0.005721 | 20.5816 |
| 3.35E-06 | 16 | 10829272 | 3579 | -0.1151 | 0.02473 | ebi-a-GCST90001624 | rs7405251 | G | T | 0.6014 | HLA DR+ CD4+ AC | TRUE | reported | igd | 0.006016 | 21.65009 |
| 1.40E-25 | 16 | 10970902 | 3579 | -0.2926 | 0.02777 | ebi-a-GCST90001624 | rs3087456 | A | G | 0.766 | HLA DR+ CD4+ AC | TRUE | reported | igd | 0.030086 | 110.9569 |
| 9.48E-06 | 19 | 56601692 | 3579 | 1.274 | 0.2872 | ebi-a-GCST90001624 | rs609755 | G | A | 0.998 | HLA DR+ CD4+ AC | TRUE | reported | igd | 0.005468 | 19.66651 |
| 2.50E-07 | 22 | 26382004 | 3579 | -0.1553 | 0.03005 | ebi-a-GCST90001624 | rs9613063 | T | C | 0.1913 | HLA DR+ CD4+ AC | TRUE | reported | igd | 0.007407 | 26.69385 |
| 4.70E-06 | 22 | 44999166 | 3579 | 0.1146 | 0.025 | ebi-a-GCST90001624 | rs12166155 | G | C | 0.3564 | HLA DR+ CD4+ AC | TRUE | reported | igd | 0.005837 | 21.00131 |
| 5.32E-06 | 2 | 53804261 | 3655 | 0.1236 | 0.02711 | ebi-a-GCST90001731 | rs10192011 | T | G | 0.29 | CD19 on IgD- CD24- | TRUE | reported | igd | 0.005655 | 20.77496 |
| 8.56E-06 | 2 | 1.5E+08 | 3655 | 0.1356 | 0.03043 | ebi-a-GCST90001731 | rs10928415 | A | G | 0.7917 | CD19 on IgD- CD24- | TRUE | reported | igd | 0.005403 | 19.84622 |
| 6.80E-06 | 3 | 60405606 | 3655 | 0.58 | 0.1287 | ebi-a-GCST90001731 | rs242172 | G | T | 0.0093 | CD19 on IgD- CD24- | TRUE | reported | igd | 0.005526 | 20.29837 |
| 9.09E-06 | 3 | 1.25E+08 | 3655 | -0.1105 | 0.02487 | ebi-a-GCST90001731 | rs631691 | T | C | 0.52 | CD19 on IgD- CD24- | TRUE | reported | igd | 0.005372 | 19.73037 |
| 2.11E-06 | 3 | 1103352 | 3655 | 0.3537 | 0.07444 | ebi-a-GCST90001731 | rs71307996 | A | G | 0.0272 | CD19 on IgD- CD24- | TRUE | reported | igd | 0.006139 | 22.56419 |
| 2.00E-06 | 4 | 1.4E+08 | 3655 | 0.2777 | 0.05834 | ebi-a-GCST90001731 | rs76451648 | C | T | 0.0469 | CD19 on IgD- CD24- | TRUE | reported | igd | 0.006161 | 22.64546 |
| 9.75E-06 | 5 | 1.13E+08 | 3655 | 0.3945 | 0.08906 | ebi-a-GCST90001731 | rs73254441 | A | G | 0.0201 | CD19 on IgD- CD24- | TRUE | reported | igd | 0.00534 | 19.6106 |
| 3.21E-06 | 6 | 75109409 | 3655 | 0.2135 | 0.04578 | ebi-a-GCST90001731 | rs16884870 | C | T | 0.0795 | CD19 on IgD- CD24- | TRUE | reported | igd | 0.005915 | 21.73734 |
| 2.78E-06 | 6 | 72550483 | 3655 | -0.8449 | 0.18 | ebi-a-GCST90001731 | rs148597489 | A | C | 0.0045 | CD19 on IgD- CD24- | TRUE | reported | igd | 0.005992 | 22.02054 |
| 9.17E-06 | 8 | 69895735 | 3655 | 0.1904 | 0.04286 | ebi-a-GCST90001731 | rs75663256 | A | C | 0.0896 | CD19 on IgD- CD24- | TRUE | reported | igd | 0.00537 | 19.72386 |
| 3.42E-06 | 10 | 11781967 | 3655 | 0.1265 | 0.0272 | ebi-a-GCST90001731 | rs10906006 | A | G | 0.7248 | CD19 on IgD- CD24- | TRUE | reported | igd | 0.005883 | 21.6175 |
| 3.71E-06 | 11 | 18601510 | 3655 | 0.8939 | 0.1929 | ebi-a-GCST90001731 | rs61884722 | C | T | 0.0041 | CD19 on IgD- CD24- | TRUE | reported | igd | 0.005841 | 21.46227 |
| 5.18E-06 | 13 | 1.12E+08 | 3655 | 0.1146 | 0.02511 | ebi-a-GCST90001731 | rs1164128 | C | T | 0.3759 | CD19 on IgD- CD24- | TRUE | reported | igd | 0.005667 | 20.81796 |
| 3.30E-06 | 13 | 41688557 | 3655 | -0.2327 | 0.04996 | ebi-a-GCST90001731 | rs7332415 | G | A | 0.0648 | CD19 on IgD- CD24- | TRUE | reported | igd | 0.005901 | 21.68254 |
| 9.53E-06 | 13 | 61899478 | 3655 | 0.8177 | 0.1844 | ebi-a-GCST90001731 | rs148027681 | C | G | 0.0044 | CD19 on IgD- CD24- | TRUE | reported | igd | 0.005351 | 19.65298 |
| 7.57E-06 | 13 | 20589957 | 3655 | -0.4372 | 0.0975 | ebi-a-GCST90001731 | rs76709166 | C | A | 0.0166 | CD19 on IgD- CD24- | TRUE | reported | igd | 0.005471 | 20.09617 |
| 7.70E-06 | 15 | 70028833 | 3655 | 0.1196 | 0.02669 | ebi-a-GCST90001731 | rs17315418 | A | G | 0.3174 | CD19 on IgD- CD24- | TRUE | reported | igd | 0.005464 | 20.06908 |
| 7.72E-06 | 16 | 28973268 | 3655 | 0.114 | 0.02546 | ebi-a-GCST90001731 | rs11643297 | G | A | 0.6112 | CD19 on IgD- CD24- | TRUE | reported | igd | 0.005455 | 20.03804 |
| 7.90E-07 | 16 | 17014673 | 3655 | -0.1879 | 0.03799 | ebi-a-GCST90001731 | rs1453509 | A | C | 0.1181 | CD19 on IgD- CD24- | TRUE | reported | igd | 0.006649 | 24.44991 |
| 1.28E-06 | 18 | 60850310 | 3655 | -0.1275 | 0.02628 | ebi-a-GCST90001731 | rs12968867 | A | G | 0.6743 | CD19 on IgD- CD24- | TRUE | reported | igd | 0.006399 | 23.52512 |
| 6.98E-06 | 19 | 5137846 | 3655 | -0.1142 | 0.02536 | ebi-a-GCST90001731 | rs2613786 | A | G | 0.3739 | CD19 on IgD- CD24- | TRUE | reported | igd | 0.005518 | 20.2673 |
| 3.82E-06 | 19 | 52235616 | 3655 | 0.2705 | 0.05845 | ebi-a-GCST90001731 | rs73056862 | T | C | 0.0461 | CD19 on IgD- CD24- | TRUE | reported | igd | 0.005826 | 21.40562 |
| 9.53E-06 | 21 | 38055091 | 3655 | -0.3513 | 0.07924 | ebi-a-GCST90001731 | rs75656751 | T | C | 0.0246 | CD19 on IgD- CD24- | TRUE | reported | igd | 0.005349 | 19.64399 |
| 4.23E-06 | 1 | 6307255 | 3656 | -0.1228 | 0.02666 | ebi-a-GCST90001732 | rs6577565 | C | T | 0.6823 | CD19 on IgD- CD27- | TRUE | reported | igd | 0.00577 | 21.20503 |
| 7.89E-06 | 1 | 27453519 | 3656 | 0.6675 | 0.1492 | ebi-a-GCST90001732 | rs113747920 | C | T | 0.0067 | CD19 on IgD- CD27- | TRUE | reported | igd | 0.005445 | 20.00448 |
| 5.23E-06 | 1 | 39551423 | 3656 | -0.1649 | 0.03615 | ebi-a-GCST90001732 | rs59102092 | C | A | 0.1368 | CD19 on IgD- CD27- | TRUE | reported | igd | 0.005659 | 20.79635 |
| 2.00E-06 | 2 | 48878727 | 3656 | 0.1482 | 0.03114 | ebi-a-GCST90001732 | rs1524155 | A | T | 0.8079 | CD19 on IgD- CD27- | TRUE | reported | igd | 0.006157 | 22.63714 |
| 7.44E-06 | 2 | 53804261 | 3656 | 0.1217 | 0.02711 | ebi-a-GCST90001732 | rs10192011 | T | G | 0.2899 | CD19 on IgD- CD27- | TRUE | reported | igd | 0.005482 | 20.14116 |
| 4.97E-06 | 2 | 2.23E+08 | 3656 | 0.1193 | 0.02609 | ebi-a-GCST90001732 | rs7608595 | C | T | 0.6675 | CD19 on IgD- CD27- | TRUE | reported | igd | 0.005687 | 20.89754 |
| 1.14E-06 | 3 | 1066077 | 3656 | 0.3581 | 0.07346 | ebi-a-GCST90001732 | rs34205222 | A | G | 0.0279 | CD19 on IgD- CD27- | TRUE | reported | igd | 0.006458 | 23.7503 |
| 2.14E-06 | 3 | 60405606 | 3656 | 0.6101 | 0.1285 | ebi-a-GCST90001732 | rs242172 | G | T | 0.0093 | CD19 on IgD- CD27- | TRUE | reported | igd | 0.006128 | 22.52984 |
| 8.12E-06 | 3 | 1.01E+08 | 3656 | 0.1142 | 0.02557 | ebi-a-GCST90001732 | rs12636323 | T | G | 0.3952 | CD19 on IgD- CD27- | TRUE | reported | igd | 0.005426 | 19.93577 |
| 5.36E-06 | 3 | 1.25E+08 | 3656 | -0.1133 | 0.02486 | ebi-a-GCST90001732 | rs631691 | T | C | 0.5201 | CD19 on IgD- CD27- | TRUE | reported | igd | 0.005649 | 20.75965 |
| 1.44E-06 | 4 | 1.73E+08 | 3656 | 0.414 | 0.08576 | ebi-a-GCST90001732 | rs115952864 | A | G | 0.0208 | CD19 on IgD- CD27- | TRUE | reported | igd | 0.006334 | 23.29129 |
| 4.18E-06 | 4 | 2746567 | 3656 | 0.2691 | 0.05837 | ebi-a-GCST90001732 | rs116412781 | T | C | 0.0475 | CD19 on IgD- CD27- | TRUE | reported | igd | 0.00578 | 21.24273 |
| 1.64E-06 | 4 | 1.63E+08 | 3656 | 0.2567 | 0.05347 | ebi-a-GCST90001732 | rs144574165 | T | C | 0.0581 | CD19 on IgD- CD27- | TRUE | reported | igd | 0.006265 | 23.03529 |
| 5.32E-06 | 5 | 1.76E+08 | 3656 | 0.3565 | 0.07821 | ebi-a-GCST90001732 | rs142291539 | A | G | 0.0269 | CD19 on IgD- CD27- | TRUE | reported | igd | 0.005651 | 20.76619 |
| 8.95E-06 | 6 | 19794487 | 3656 | -0.2277 | 0.05119 | ebi-a-GCST90001732 | rs9350187 | T | C | 0.0607 | CD19 on IgD- CD27- | TRUE | reported | igd | 0.005383 | 19.77508 |
| 9.58E-06 | 6 | 75109171 | 3656 | 0.2028 | 0.04575 | ebi-a-GCST90001732 | rs71555251 | A | G | 0.0796 | CD19 on IgD- CD27- | TRUE | reported | igd | 0.005346 | 19.63885 |
| 8.02E-06 | 7 | 51994027 | 3656 | -0.1259 | 0.02817 | ebi-a-GCST90001732 | rs62463053 | C | G | 0.2629 | CD19 on IgD- CD27- | TRUE | reported | igd | 0.005434 | 19.96366 |
| 3.91E-06 | 8 | 69895735 | 3656 | 0.1981 | 0.04284 | ebi-a-GCST90001732 | rs75663256 | A | C | 0.0896 | CD19 on IgD- CD27- | TRUE | reported | igd | 0.005815 | 21.37137 |
| 6.26E-06 | 8 | 1.43E+08 | 3656 | -0.1112 | 0.02458 | ebi-a-GCST90001732 | rs10216993 | T | C | 0.4624 | CD19 on IgD- CD27- | TRUE | reported | igd | 0.005567 | 20.45541 |
| 2.73E-06 | 9 | 95896099 | 3656 | -0.1158 | 0.02466 | ebi-a-GCST90001732 | rs10821075 | A | G | 0.4393 | CD19 on IgD- CD27- | TRUE | reported | igd | 0.005995 | 22.03907 |
| 3.18E-06 | 11 | 18601510 | 3656 | 0.8987 | 0.1926 | ebi-a-GCST90001732 | rs61884722 | C | T | 0.0041 | CD19 on IgD- CD27- | TRUE | reported | igd | 0.00592 | 21.76102 |
| 5.27E-06 | 13 | 20089809 | 3656 | -0.4166 | 0.09136 | ebi-a-GCST90001732 | rs148390689 | C | T | 0.0186 | CD19 on IgD- CD27- | TRUE | reported | igd | 0.005655 | 20.78207 |
| 6.80E-06 | 15 | 45169337 | 3656 | -0.2039 | 0.04525 | ebi-a-GCST90001732 | rs114723901 | A | G | 0.0873 | CD19 on IgD- CD27- | TRUE | reported | igd | 0.005523 | 20.29363 |
| 5.23E-06 | 16 | 28973268 | 3656 | 0.1162 | 0.02547 | ebi-a-GCST90001732 | rs11643297 | G | A | 0.6111 | CD19 on IgD- CD27- | TRUE | reported | igd | 0.005661 | 20.80256 |
| 4.54E-07 | 16 | 32422808 | 3656 | -1.105 | 0.2187 | ebi-a-GCST90001732 | rs138920008 | C | G | 0.0041 | CD19 on IgD- CD27- | TRUE | reported | igd | 0.006934 | 25.51463 |
| 1.92E-07 | 18 | 60850310 | 3656 | -0.137 | 0.02626 | ebi-a-GCST90001732 | rs12968867 | A | G | 0.6744 | CD19 on IgD- CD27- | TRUE | reported | igd | 0.00739 | 27.20283 |
| 3.85E-06 | 22 | 43167446 | 3656 | -0.2905 | 0.06279 | ebi-a-GCST90001732 | rs113612962 | C | G | 0.0402 | CD19 on IgD- CD27- | TRUE | reported | igd | 0.005821 | 21.3931 |
| 2.27E-06 | 1 | 1.95E+08 | 3656 | -0.1554 | 0.03281 | ebi-a-GCST90001811 | rs12032479 | T | C | 0.1797 | CD38 on IgD+ CD24- | TRUE | reported | igd | 0.006099 | 22.42084 |
| 1.60E-06 | 3 | 1.28E+08 | 3656 | 0.9983 | 0.2077 | ebi-a-GCST90001811 | rs142962704 | T | C | 0.0034 | CD38 on IgD+ CD24- | TRUE | reported | igd | 0.006279 | 23.08934 |
| 7.10E-06 | 3 | 14288322 | 3656 | 0.3949 | 0.08781 | ebi-a-GCST90001811 | rs79300919 | A | G | 0.0211 | CD38 on IgD+ CD24- | TRUE | reported | igd | 0.005502 | 20.21383 |
| 1.89E-06 | 4 | 14029370 | 3656 | -0.3492 | 0.07317 | ebi-a-GCST90001811 | rs572542 | T | C | 0.0289 | CD38 on IgD+ CD24- | TRUE | reported | igd | 0.006191 | 22.7638 |
| 4.58E-18 | 4 | 15915069 | 3656 | -0.7826 | 0.08987 | ebi-a-GCST90001811 | rs76112278 | A | G | 0.0194 | CD38 on IgD+ CD24- | TRUE | reported | igd | 0.02032 | 75.79011 |
| 8.99E-06 | 4 | 1.57E+08 | 3656 | 0.3099 | 0.0697 | ebi-a-GCST90001811 | rs28393044 | G | C | 0.0334 | CD38 on IgD+ CD24- | TRUE | reported | igd | 0.005378 | 19.75786 |
| 7.97E-09 | 4 | 15956227 | 3656 | -0.6992 | 0.1209 | ebi-a-GCST90001811 | rs115062208 | G | A | 0.0115 | CD38 on IgD+ CD24- | TRUE | reported | igd | 0.009065 | 33.42817 |
| 2.48E-07 | 4 | 15037408 | 3656 | -0.3606 | 0.06977 | ebi-a-GCST90001811 | rs76354299 | A | G | 0.0321 | CD38 on IgD+ CD24- | TRUE | reported | igd | 0.007253 | 26.69785 |
| 2.60E-06 | 5 | 91363908 | 3656 | -0.1644 | 0.03492 | ebi-a-GCST90001811 | rs6895789 | T | C | 0.1533 | CD38 on IgD+ CD24- | TRUE | reported | igd | 0.006026 | 22.15223 |
| 1.05E-06 | 6 | 30228721 | 3656 | 0.1331 | 0.02723 | ebi-a-GCST90001811 | rs2516708 | A | G | 0.5252 | CD38 on IgD+ CD24- | TRUE | reported | igd | 0.006493 | 23.87939 |
| 4.21E-06 | 7 | 3020375 | 3656 | -0.1139 | 0.02471 | ebi-a-GCST90001811 | rs1878808 | T | C | 0.4721 | CD38 on IgD+ CD24- | TRUE | reported | igd | 0.005778 | 21.23559 |
| 7.54E-06 | 9 | 15034791 | 3656 | -0.4872 | 0.1087 | ebi-a-GCST90001811 | rs72715691 | T | A | 0.0131 | CD38 on IgD+ CD24- | TRUE | reported | igd | 0.005465 | 20.07788 |
| 1.09E-06 | 11 | 8825966 | 3656 | 0.1233 | 0.02525 | ebi-a-GCST90001811 | rs11042077 | A | G | 0.4943 | CD38 on IgD+ CD24- | TRUE | reported | igd | 0.00648 | 23.83229 |
| 2.83E-06 | 13 | 72594895 | 3656 | 0.1395 | 0.02975 | ebi-a-GCST90001811 | rs9599916 | G | A | 0.2291 | CD38 on IgD+ CD24- | TRUE | reported | igd | 0.005978 | 21.9754 |
| 2.45E-06 | 14 | 38174359 | 3656 | 0.1195 | 0.02533 | ebi-a-GCST90001811 | rs17179337 | G | A | 0.4724 | CD38 on IgD+ CD24- | TRUE | reported | igd | 0.006051 | 22.24476 |
| 9.86E-06 | 14 | 87885710 | 3656 | -0.537 | 0.1213 | ebi-a-GCST90001811 | rs147970068 | T | G | 0.0107 | CD38 on IgD+ CD24- | TRUE | reported | igd | 0.005332 | 19.58797 |
| 6.35E-06 | 20 | 12142943 | 3656 | -0.7342 | 0.1624 | ebi-a-GCST90001811 | rs78111008 | C | T | 0.0063 | CD38 on IgD+ CD24- | TRUE | reported | igd | 0.005559 | 20.42768 |
| 7.52E-06 | 1 | 32615715 | 2920 | 0.6661 | 0.1485 | ebi-a-GCST90001894 | rs138089143 | C | G | 0.0079 | CD28 on CD28+ CD4+ | TRUE | reported | igd | 0.006843 | 20.10613 |
| 2.04E-06 | 2 | 2.04E+08 | 2920 | -0.2223 | 0.04671 | ebi-a-GCST90001894 | rs79296624 | C | A | 0.0978 | CD28 on CD28+ CD4+ | TRUE | reported | igd | 0.007697 | 22.63402 |
| 2.88E-06 | 2 | 20856144 | 2920 | -0.249 | 0.0531 | ebi-a-GCST90001894 | rs13023055 | A | G | 0.0705 | CD28 on CD28+ CD4+ | TRUE | reported | igd | 0.007474 | 21.97415 |
| 2.24E-42 | 2 | 2.05E+08 | 2920 | 0.3858 | 0.02783 | ebi-a-GCST90001894 | rs1973872 | G | T | 0.601 | CD28 on CD28+ CD4+ | TRUE | reported | igd | 0.06175 | 192.0439 |
| 6.40E-06 | 3 | 1.39E+08 | 2920 | -0.1998 | 0.0442 | ebi-a-GCST90001894 | rs360711 | G | T | 0.8911 | CD28 on CD28+ CD4+ | TRUE | reported | igd | 0.006949 | 20.41968 |
| 8.42E-06 | 4 | 8533012 | 2920 | 0.1263 | 0.02831 | ebi-a-GCST90001894 | rs7685596 | G | A | 0.3664 | CD28 on CD28+ CD4+ | TRUE | reported | igd | 0.00677 | 19.88975 |
| 2.56E-06 | 4 | 13704839 | 2920 | -0.2738 | 0.05811 | ebi-a-GCST90001894 | rs115478283 | G | A | 0.057 | CD28 on CD28+ CD4+ | TRUE | reported | igd | 0.007546 | 22.18542 |
| 1.17E-06 | 4 | 24489778 | 2920 | 0.1936 | 0.03974 | ebi-a-GCST90001894 | rs116298583 | T | A | 0.1384 | CD28 on CD28+ CD4+ | TRUE | reported | igd | 0.008062 | 23.71687 |
| 1.14E-06 | 4 | 29416600 | 2920 | 0.2933 | 0.06015 | ebi-a-GCST90001894 | rs6836990 | T | G | 0.0572 | CD28 on CD28+ CD4+ | TRUE | reported | igd | 0.008077 | 23.76048 |
| 2.81E-07 | 5 | 5367152 | 2920 | 1.949 | 0.3787 | ebi-a-GCST90001894 | rs77397796 | T | C | 0.0012 | CD28 on CD28+ CD4+ | TRUE | reported | igd | 0.008989 | 26.46888 |
| 8.93E-07 | 5 | 1.25E+08 | 2920 | -0.4929 | 0.1001 | ebi-a-GCST90001894 | rs59159369 | G | T | 0.0193 | CD28 on CD28+ CD4+ | TRUE | reported | igd | 0.008235 | 24.22992 |
| 9.80E-06 | 6 | 29878483 | 2920 | -0.1396 | 0.03152 | ebi-a-GCST90001894 | rs3128990 | T | C | 0.5247 | CD28 on CD28+ CD4+ | TRUE | reported | igd | 0.006673 | 19.60202 |
| 1.84E-13 | 6 | 90976768 | 2920 | 0.2512 | 0.03397 | ebi-a-GCST90001894 | rs72928038 | A | G | 0.199 | CD28 on CD28+ CD4+ | TRUE | reported | igd | 0.018383 | 54.64502 |
| 3.47E-06 | 6 | 1.55E+08 | 2920 | -0.145 | 0.03118 | ebi-a-GCST90001894 | rs6557390 | G | A | 0.7538 | CD28 on CD28+ CD4+ | TRUE | reported | igd | 0.007352 | 21.61157 |
| 3.52E-06 | 7 | 43252572 | 2920 | -0.8432 | 0.1814 | ebi-a-GCST90001894 | rs62458220 | C | T | 0.006 | CD28 on CD28+ CD4+ | TRUE | reported | igd | 0.007345 | 21.59181 |
| 7.80E-07 | 8 | 72575417 | 2920 | 0.1763 | 0.0356 | ebi-a-GCST90001894 | rs55637775 | A | G | 0.1815 | CD28 on CD28+ CD4+ | TRUE | reported | igd | 0.008329 | 24.50795 |
| 9.22E-06 | 8 | 1.38E+08 | 2920 | -0.3439 | 0.0774 | ebi-a-GCST90001894 | rs111970139 | G | A | 0.0324 | CD28 on CD28+ CD4+ | TRUE | reported | igd | 0.006715 | 19.72808 |
| 9.92E-06 | 9 | 27088757 | 2920 | -0.1672 | 0.03778 | ebi-a-GCST90001894 | rs62542710 | G | A | 0.1582 | CD28 on CD28+ CD4+ | TRUE | reported | igd | 0.006663 | 19.57272 |
| 7.72E-06 | 10 | 1.15E+08 | 2920 | -2.331 | 0.5201 | ebi-a-GCST90001894 | rs142599237 | T | C | 7.00E-04 | CD28 on CD28+ CD4+ | TRUE | reported | igd | 0.006832 | 20.07305 |
| 8.78E-06 | 11 | 56654147 | 2920 | -0.4003 | 0.0899 | ebi-a-GCST90001894 | rs17640797 | A | G | 0.0233 | CD28 on CD28+ CD4+ | TRUE | reported | igd | 0.006744 | 19.81318 |
| 6.69E-06 | 11 | 1.34E+08 | 2920 | 0.1222 | 0.02709 | ebi-a-GCST90001894 | rs3133072 | C | T | 0.5158 | CD28 on CD28+ CD4+ | TRUE | reported | igd | 0.00692 | 20.33419 |
| 9.21E-06 | 12 | 1.31E+08 | 2920 | -0.2773 | 0.06242 | ebi-a-GCST90001894 | rs11060804 | A | G | 0.0509 | CD28 on CD28+ CD4+ | TRUE | reported | igd | 0.006713 | 19.72217 |
| 4.88E-06 | 13 | 1.14E+08 | 2920 | 0.2676 | 0.05846 | ebi-a-GCST90001894 | rs117505260 | T | G | 0.0563 | CD28 on CD28+ CD4+ | TRUE | reported | igd | 0.007125 | 20.93905 |
| 3.85E-06 | 13 | 97958649 | 2920 | 0.3399 | 0.07344 | ebi-a-GCST90001894 | rs9516901 | C | T | 0.0353 | CD28 on CD28+ CD4+ | TRUE | reported | igd | 0.007282 | 21.40619 |
| 8.34E-06 | 14 | 57274519 | 2920 | 0.1358 | 0.03042 | ebi-a-GCST90001894 | rs1123285 | G | C | 0.2844 | CD28 on CD28+ CD4+ | TRUE | reported | igd | 0.006779 | 19.91515 |
| 5.71E-07 | 15 | 70299168 | 2920 | -0.2056 | 0.04102 | ebi-a-GCST90001894 | rs16954045 | T | C | 0.1279 | CD28 on CD28+ CD4+ | TRUE | reported | igd | 0.00853 | 25.10483 |
| 5.76E-06 | 21 | 16860484 | 2920 | 0.303 | 0.06668 | ebi-a-GCST90001894 | rs998490 | T | G | 0.043 | CD28 on CD28+ CD4+ | TRUE | reported | igd | 0.007022 | 20.63462 |
| 8.94E-07 | 22 | 24996630 | 2920 | 0.1429 | 0.02903 | ebi-a-GCST90001894 | rs5751902 | T | C | 0.3435 | CD28 on CD28+ CD4+ | TRUE | reported | igd | 0.00823 | 24.21435 |
| 9.01E-07 | 1 | 3272937 | 2650 | 1.078 | 0.2189 | ebi-a-GCST90001901 | rs141247369 | A | G | 0.0038 | CD28 on CD39+ resting Treg | TRUE | reported | igd | 0.009069 | 24.23361 |
| 1.71E-06 | 1 | 1.77E+08 | 2650 | 0.7664 | 0.1598 | ebi-a-GCST90001901 | rs114565907 | T | G | 0.0074 | CD28 on CD39+ resting Treg | TRUE | reported | igd | 0.008605 | 22.98421 |
| 3.99E-06 | 1 | 6127983 | 2650 | -0.1602 | 0.03467 | ebi-a-GCST90001901 | rs12068559 | C | T | 0.8096 | CD28 on CD39+ resting Treg | TRUE | reported | igd | 0.007993 | 21.33484 |
| 8.40E-06 | 1 | 2.42E+08 | 2650 | -4.226 | 0.9469 | ebi-a-GCST90001901 | rs115841111 | G | A | 2.00E-04 | CD28 on CD39+ resting Treg | TRUE | reported | igd | 0.00746 | 19.9032 |
| 1.87E-07 | 2 | 2.05E+08 | 2650 | -0.1787 | 0.0342 | ebi-a-GCST90001901 | rs13404978 | T | C | 0.1977 | CD28 on CD39+ resting Treg | TRUE | reported | igd | 0.010198 | 27.28155 |
| 8.97E-06 | 2 | 39471924 | 2650 | -0.1197 | 0.0269 | ebi-a-GCST90001901 | rs12614511 | G | A | 0.5285 | CD28 on CD39+ resting Treg | TRUE | reported | igd | 0.007417 | 19.7859 |
| 6.49E-06 | 4 | 1.29E+08 | 2650 | -2.595 | 0.5743 | ebi-a-GCST90001901 | rs114550156 | T | C | 6.00E-04 | CD28 on CD39+ resting Treg | TRUE | reported | igd | 0.007646 | 20.40183 |
| 4.50E-06 | 6 | 13328984 | 2650 | 0.4675 | 0.1017 | ebi-a-GCST90001901 | rs78365755 | C | G | 0.0177 | CD28 on CD39+ resting Treg | TRUE | reported | igd | 0.007911 | 21.11511 |
| 3.44E-09 | 6 | 90946479 | 2650 | 0.1619 | 0.0273 | ebi-a-GCST90001901 | rs4707609 | C | T | 0.4387 | CD28 on CD39+ resting Treg | TRUE | reported | igd | 0.013098 | 35.14314 |
| 2.94E-06 | 6 | 1.38E+08 | 2650 | -1.533 | 0.3271 | ebi-a-GCST90001901 | rs623219 | T | C | 0.9983 | CD28 on CD39+ resting Treg | TRUE | reported | igd | 0.00822 | 21.94802 |
| 3.35E-06 | 8 | 1.44E+08 | 2650 | 0.1273 | 0.02734 | ebi-a-GCST90001901 | rs7846567 | A | C | 0.5181 | CD28 on CD39+ resting Treg | TRUE | reported | igd | 0.008115 | 21.66366 |
| 2.28E-06 | 10 | 96597621 | 2650 | 0.4033 | 0.08514 | ebi-a-GCST90001901 | rs117957417 | A | G | 0.0257 | CD28 on CD39+ resting Treg | TRUE | reported | igd | 0.008396 | 22.42132 |
| 5.89E-06 | 10 | 61151603 | 2650 | 0.136 | 0.02997 | ebi-a-GCST90001901 | rs284615 | G | A | 0.2915 | CD28 on CD39+ resting Treg | TRUE | reported | igd | 0.007711 | 20.57673 |
| 2.67E-06 | 10 | 1.22E+08 | 2650 | -2.668 | 0.567 | ebi-a-GCST90001901 | rs118122984 | G | A | 6.00E-04 | CD28 on CD39+ resting Treg | TRUE | reported | igd | 0.008286 | 22.12471 |
| 9.29E-12 | 10 | 97551952 | 2650 | 0.4455 | 0.06505 | ebi-a-GCST90001901 | rs117653391 | A | G | 0.0449 | CD28 on CD39+ resting Treg | TRUE | reported | igd | 0.017391 | 46.86762 |
| 1.45E-06 | 11 | 37444749 | 2650 | 1.149 | 0.2378 | ebi-a-GCST90001901 | rs138199636 | T | C | 0.0036 | CD28 on CD39+ resting Treg | TRUE | reported | igd | 0.008733 | 23.32859 |
| 6.54E-07 | 12 | 70389055 | 2650 | -0.2152 | 0.04315 | ebi-a-GCST90001901 | rs17107510 | T | C | 0.1164 | CD28 on CD39+ resting Treg | TRUE | reported | igd | 0.009299 | 24.85393 |
| 1.58E-07 | 13 | 80391747 | 2650 | 0.9895 | 0.1882 | ebi-a-GCST90001901 | rs182187758 | G | C | 0.0053 | CD28 on CD39+ resting Treg | TRUE | reported | igd | 0.010324 | 27.62259 |
| 9.35E-06 | 14 | 48325261 | 2650 | 0.122 | 0.02748 | ebi-a-GCST90001901 | rs447600 | A | T | 0.5174 | CD28 on CD39+ resting Treg | TRUE | reported | igd | 0.007383 | 19.69511 |
| 9.11E-08 | 15 | 87662869 | 2650 | -0.2975 | 0.05552 | ebi-a-GCST90001901 | rs114579738 | T | G | 0.0626 | CD28 on CD39+ resting Treg | TRUE | reported | igd | 0.010719 | 28.6911 |
| 7.22E-06 | 16 | 634475 | 2650 | 0.3459 | 0.07694 | ebi-a-GCST90001901 | rs117389073 | T | C | 0.0319 | CD28 on CD39+ resting Treg | TRUE | reported | igd | 0.007569 | 20.19616 |
| 1.37E-06 | 17 | 11968129 | 2650 | 0.2663 | 0.05502 | ebi-a-GCST90001901 | rs28921682 | A | G | 0.0649 | CD28 on CD39+ resting Treg | TRUE | reported | igd | 0.008763 | 23.40848 |
| 1.51E-06 | 22 | 48371416 | 2650 | -0.2399 | 0.04977 | ebi-a-GCST90001901 | rs6008485 | G | A | 0.0808 | CD28 on CD39+ resting Treg | TRUE | reported | igd | 0.008691 | 23.21653 |
| 6.93E-06 | 1 | 77233774 | 2872 | -0.1237 | 0.02747 | ebi-a-GCST90002072 | rs12410542 | T | C | 0.5218 | SSC-A on plasmacytoid DC | TRUE | reported | igd | 0.007011 | 20.26374 |
| 4.94E-06 | 1 | 1.02E+08 | 2872 | 0.8331 | 0.1821 | ebi-a-GCST90002072 | rs11164360 | C | T | 0.0061 | SSC-A on plasmacytoid DC | TRUE | reported | igd | 0.007235 | 20.91567 |
| 1.27E-06 | 2 | 1.37E+08 | 2872 | 0.1493 | 0.03074 | ebi-a-GCST90002072 | rs10193587 | C | T | 0.7214 | SSC-A on plasmacytoid DC | TRUE | reported | igd | 0.008147 | 23.5727 |
| 6.31E-06 | 2 | 2.43E+08 | 2872 | 0.2975 | 0.06577 | ebi-a-GCST90002072 | rs71423107 | A | G | 0.0679 | SSC-A on plasmacytoid DC | TRUE | reported | igd | 0.007074 | 20.44635 |
| 3.98E-07 | 2 | 1.69E+08 | 2872 | 0.1412 | 0.02779 | ebi-a-GCST90002072 | rs2084848 | C | G | 0.5787 | SSC-A on plasmacytoid DC | TRUE | reported | igd | 0.008909 | 25.79822 |
| 4.31E-14 | 4 | 84157443 | 2872 | -0.2195 | 0.02892 | ebi-a-GCST90002072 | rs2903918 | G | C | 0.3407 | SSC-A on plasmacytoid DC | TRUE | reported | igd | 0.019664 | 57.56651 |
| 6.69E-06 | 5 | 74298665 | 2872 | -0.1812 | 0.04016 | ebi-a-GCST90002072 | rs13162242 | G | T | 0.1382 | SSC-A on plasmacytoid DC | TRUE | reported | igd | 0.007038 | 20.34354 |
| 6.41E-06 | 6 | 95562104 | 2872 | -0.4324 | 0.09564 | ebi-a-GCST90002072 | rs79711662 | T | G | 0.0207 | SSC-A on plasmacytoid DC | TRUE | reported | igd | 0.007067 | 20.4263 |
| 2.54E-06 | 6 | 84970341 | 2872 | 0.3189 | 0.06764 | ebi-a-GCST90002072 | rs17791425 | A | G | 0.0442 | SSC-A on plasmacytoid DC | TRUE | reported | igd | 0.00768 | 22.2126 |
| 8.95E-06 | 7 | 24079081 | 2872 | -0.3673 | 0.08255 | ebi-a-GCST90002072 | rs80067671 | C | T | 0.0286 | SSC-A on plasmacytoid DC | TRUE | reported | igd | 0.006846 | 19.78359 |
| 1.20E-08 | 7 | 50318308 | 2872 | -0.1851 | 0.03237 | ebi-a-GCST90002072 | rs17552904 | T | G | 0.245 | SSC-A on plasmacytoid DC | TRUE | reported | igd | 0.011257 | 32.6757 |
| 8.65E-06 | 8 | 1.18E+08 | 2872 | -0.1298 | 0.02913 | ebi-a-GCST90002072 | rs6987643 | C | T | 0.3249 | SSC-A on plasmacytoid DC | TRUE | reported | igd | 0.006866 | 19.84111 |
| 9.57E-06 | 10 | 42747410 | 2872 | -0.4799 | 0.1082 | ebi-a-GCST90002072 | rs537539184 | T | C | 0.0183 | SSC-A on plasmacytoid DC | TRUE | reported | igd | 0.006803 | 19.65823 |
| 1.81E-08 | 11 | 4960640 | 2872 | -0.3341 | 0.05919 | ebi-a-GCST90002072 | rs142456232 | T | C | 0.0573 | SSC-A on plasmacytoid DC | TRUE | reported | igd | 0.010972 | 31.83858 |
| 4.41E-08 | 11 | 5544477 | 2872 | -0.4036 | 0.07353 | ebi-a-GCST90002072 | rs78736654 | G | T | 0.0378 | SSC-A on plasmacytoid DC | TRUE | reported | igd | 0.010381 | 30.10722 |
| 6.30E-06 | 12 | 70018656 | 2872 | 0.1536 | 0.03394 | ebi-a-GCST90002072 | rs776409 | G | T | 0.2162 | SSC-A on plasmacytoid DC | TRUE | reported | igd | 0.007081 | 20.4671 |
| 2.94E-06 | 12 | 52279540 | 2872 | -0.5379 | 0.1148 | ebi-a-GCST90002072 | rs9669659 | A | G | 0.0146 | SSC-A on plasmacytoid DC | TRUE | reported | igd | 0.007586 | 21.939 |
| 1.68E-06 | 14 | 1.02E+08 | 2872 | -0.1866 | 0.03888 | ebi-a-GCST90002072 | rs8022420 | A | G | 0.8518 | SSC-A on plasmacytoid DC | TRUE | reported | igd | 0.007956 | 23.01803 |
| 2.27E-06 | 16 | 57726123 | 2872 | 0.1539 | 0.03247 | ebi-a-GCST90002072 | rs2923129 | A | G | 0.2274 | SSC-A on plasmacytoid DC | TRUE | reported | igd | 0.007761 | 22.44968 |
| 9.86E-06 | 16 | 4436279 | 2872 | -2.137 | 0.4826 | ebi-a-GCST90002072 | rs71388573 | G | T | 9.00E-04 | SSC-A on plasmacytoid DC | TRUE | reported | igd | 0.006781 | 19.5944 |
| 3.19E-07 | 16 | 89141723 | 2872 | -0.1957 | 0.03819 | ebi-a-GCST90002072 | rs55733613 | C | G | 0.1628 | SSC-A on plasmacytoid DC | TRUE | reported | igd | 0.00906 | 26.24096 |
| 8.57E-06 | 16 | 74142321 | 2872 | 0.1566 | 0.03513 | ebi-a-GCST90002072 | rs6564921 | A | T | 0.8048 | SSC-A on plasmacytoid DC | TRUE | reported | igd | 0.006871 | 19.8575 |
| 2.78E-06 | 16 | 60631203 | 2872 | 0.1344 | 0.02863 | ebi-a-GCST90002072 | rs6500136 | A | G | 0.3917 | SSC-A on plasmacytoid DC | TRUE | reported | igd | 0.007615 | 22.02182 |
| 4.15E-07 | 17 | 62128083 | 2872 | 0.4562 | 0.08992 | ebi-a-GCST90002072 | rs117067704 | G | A | 0.0232 | SSC-A on plasmacytoid DC | TRUE | reported | igd | 0.008883 | 25.72145 |
| 1.78E-06 | 17 | 63066415 | 2872 | 0.2991 | 0.06248 | ebi-a-GCST90002072 | rs144843975 | T | C | 0.0519 | SSC-A on plasmacytoid DC | TRUE | reported | igd | 0.007916 | 22.90067 |
| 3.49E-09 | 17 | 8677215 | 2872 | 0.1642 | 0.02772 | ebi-a-GCST90002072 | rs722926 | T | C | 0.4102 | SSC-A on plasmacytoid DC | TRUE | reported | igd | 0.01207 | 35.06367 |
| 7.57E-06 | 19 | 56863306 | 2872 | 0.1289 | 0.02873 | ebi-a-GCST90002072 | rs2058319 | G | A | 0.652 | SSC-A on plasmacytoid DC | TRUE | reported | igd | 0.00696 | 20.11556 |
| 5.99E-06 | 21 | 34218354 | 2872 | -0.3578 | 0.0789 | ebi-a-GCST90002072 | rs144283125 | G | A | 0.0312 | SSC-A on plasmacytoid DC | TRUE | reported | igd | 0.00711 | 20.55058 |
| 1.43E-38 | 1 | 1.61E+08 | 1578 | 0.4941 | 0.03702 | ebi-a-GCST90002097 | rs1801274 | G | A | 0.3305 | CD11b on CD33br HLA DR+ CD14dim | TRUE | reported | igd | 0.101437 | 177.9123 |
| 9.69E-06 | 2 | 2.01E+08 | 1578 | 0.3891 | 0.08767 | ebi-a-GCST90002097 | rs76769365 | C | G | 0.0428 | CD11b on CD33br HLA DR+ CD14dim | TRUE | reported | igd | 0.012329 | 19.67296 |
| 4.95E-06 | 3 | 1.19E+08 | 1578 | -1.066 | 0.2325 | ebi-a-GCST90002097 | rs142436127 | A | T | 0.0067 | CD11b on CD33br HLA DR+ CD14dim | TRUE | reported | igd | 0.013147 | 20.99509 |
| 3.02E-06 | 4 | 55977759 | 1578 | -0.166 | 0.03541 | ebi-a-GCST90002097 | rs3943404 | T | A | 0.481 | CD11b on CD33br HLA DR+ CD14dim | TRUE | reported | igd | 0.013736 | 21.94894 |
| 6.88E-06 | 7 | 55375099 | 1578 | 0.6737 | 0.1493 | ebi-a-GCST90002097 | rs142470748 | A | G | 0.0143 | CD11b on CD33br HLA DR+ CD14dim | TRUE | reported | igd | 0.012739 | 20.33587 |
| 3.44E-07 | 7 | 92609625 | 1578 | 0.3209 | 0.06268 | ebi-a-GCST90002097 | rs79788679 | G | A | 0.083 | CD11b on CD33br HLA DR+ CD14dim | TRUE | reported | igd | 0.016339 | 26.17765 |
| 9.71E-07 | 7 | 42123925 | 1578 | 0.1828 | 0.03718 | ebi-a-GCST90002097 | rs846294 | C | G | 0.3213 | CD11b on CD33br HLA DR+ CD14dim | TRUE | reported | igd | 0.015088 | 24.14253 |
| 6.42E-06 | 9 | 87310577 | 1578 | 0.3263 | 0.07207 | ebi-a-GCST90002097 | rs62561188 | C | T | 0.0669 | CD11b on CD33br HLA DR+ CD14dim | TRUE | reported | igd | 0.012824 | 20.47266 |
| 6.67E-06 | 9 | 1.01E+08 | 1578 | -0.3105 | 0.06871 | ebi-a-GCST90002097 | rs1930423 | A | T | 0.0713 | CD11b on CD33br HLA DR+ CD14dim | TRUE | reported | igd | 0.012776 | 20.39541 |
| 3.23E-06 | 10 | 26677896 | 1578 | -0.172 | 0.03681 | ebi-a-GCST90002097 | rs11015080 | T | A | 0.5856 | CD11b on CD33br HLA DR+ CD14dim | TRUE | reported | igd | 0.013647 | 21.80592 |
| 9.73E-06 | 10 | 9580495 | 1578 | -0.2427 | 0.05469 | ebi-a-GCST90002097 | rs66811768 | T | G | 0.1188 | CD11b on CD33br HLA DR+ CD14dim | TRUE | reported | igd | 0.012326 | 19.66858 |
| 7.64E-06 | 12 | 1.33E+08 | 1578 | -0.2557 | 0.05696 | ebi-a-GCST90002097 | rs61952076 | A | G | 0.1119 | CD11b on CD33br HLA DR+ CD14dim | TRUE | reported | igd | 0.01261 | 20.12661 |
| 9.32E-06 | 12 | 218152 | 1578 | -0.4766 | 0.1072 | ebi-a-GCST90002097 | rs57761043 | A | G | 0.0292 | CD11b on CD33br HLA DR+ CD14dim | TRUE | reported | igd | 0.012371 | 19.74094 |
| 6.75E-06 | 12 | 1.13E+08 | 1578 | 0.1612 | 0.03568 | ebi-a-GCST90002097 | rs10850001 | A | T | 0.5304 | CD11b on CD33br HLA DR+ CD14dim | TRUE | reported | igd | 0.01277 | 20.38589 |
| 7.04E-07 | 13 | 74097996 | 1578 | -0.8413 | 0.1689 | ebi-a-GCST90002097 | rs140129370 | C | T | 0.0114 | CD11b on CD33br HLA DR+ CD14dim | TRUE | reported | igd | 0.01548 | 24.77945 |
| 8.81E-07 | 14 | 73295385 | 1578 | 1.104 | 0.2236 | ebi-a-GCST90002097 | rs146147816 | A | G | 0.0067 | CD11b on CD33br HLA DR+ CD14dim | TRUE | reported | igd | 0.015214 | 24.34691 |
| 2.01E-06 | 15 | 51443825 | 1578 | 0.3401 | 0.07129 | ebi-a-GCST90002097 | rs75948515 | T | G | 0.0703 | CD11b on CD33br HLA DR+ CD14dim | TRUE | reported | igd | 0.014218 | 22.7303 |
| 4.77E-06 | 15 | 84637928 | 1578 | 0.1643 | 0.03578 | ebi-a-GCST90002097 | rs7166641 | T | C | 0.5957 | CD11b on CD33br HLA DR+ CD14dim | TRUE | reported | igd | 0.013186 | 21.05929 |
| 9.28E-06 | 16 | 83304908 | 1578 | -0.2884 | 0.06484 | ebi-a-GCST90002097 | rs80184675 | A | G | 0.0887 | CD11b on CD33br HLA DR+ CD14dim | TRUE | reported | igd | 0.012382 | 19.75849 |
| 5.30E-06 | 17 | 11788269 | 1578 | 0.7221 | 0.1581 | ebi-a-GCST90002097 | rs72815426 | G | A | 0.0124 | CD11b on CD33br HLA DR+ CD14dim | TRUE | reported | igd | 0.013047 | 20.83436 |
| 7.47E-06 | 17 | 75786314 | 1578 | 0.1631 | 0.03629 | ebi-a-GCST90002097 | rs55978635 | G | C | 0.3606 | CD11b on CD33br HLA DR+ CD14dim | TRUE | reported | igd | 0.012639 | 20.17359 |
| 2.52E-06 | 18 | 38747705 | 1578 | 0.2014 | 0.04263 | ebi-a-GCST90002097 | rs2552498 | C | T | 0.7706 | CD11b on CD33br HLA DR+ CD14dim | TRUE | reported | igd | 0.013947 | 22.29141 |
| 5.05E-06 | 18 | 75884528 | 1578 | -0.3751 | 0.08192 | ebi-a-GCST90002097 | rs72983710 | T | C | 0.0504 | CD11b on CD33br HLA DR+ CD14dim | TRUE | reported | igd | 0.013112 | 20.93936 |
